# Bayesian change-point modeling with segmented ARMA model

**DOI:** 10.1371/journal.pone.0208927

**Published:** 2018-12-31

**Authors:** Farhana Sadia, Sarah Boyd, Jonathan M. Keith

**Affiliations:** 1 School of Mathematical Sciences, Monash University, Clayton, VIC, Australia; 2 Faculty of Information Technology, Monash University, Clayton, VIC, Australia; University of Bath, UNITED KINGDOM

## Abstract

Time series segmentation aims to identify segment boundary points in a time series, and to determine the dynamical properties corresponding to each segment. To segment time series data, this article presents a Bayesian change-point model in which the data within segments follows an autoregressive moving average (ARMA) model. A prior distribution is defined for the number of change-points, their positions, segment means and error terms. To quantify uncertainty about the location of change-points, the resulting posterior probability distributions are sampled using the Generalized Gibbs sampler Markov chain Monte Carlo technique. This methodology is illustrated by applying it to simulated data and to real data known as the well-log time series data. This well-log data records the measurements of nuclear magnetic response of underground rocks during the drilling of a well. Our approach has high sensitivity, and detects a larger number of change-points than have been identified by comparable methods in the existing literature.

## Introduction

A time series is a succession of measurements made over a time interval. Some time series can be divided into a sequence of individual segments, each with its own unique characteristic properties. Identifying segment boundaries and inferring dynamical properties of different segments is referred to as time series segmentation. Change-point detection methods can be used to segment time series data since the goal of such methods is to select a sequence of change-point locations such that the observations are, in some sense, homogeneous within segments and different between segments [1].

Statistical analysis of change-point problems has been the subject of intensive research in the past half-century and there has been a large amount of literature on this subject (for reviews, see [2–5]). Literature on Bayesian change-point modeling is as extensive as for the classical change-point model. The Bayesian change-point model was pioneered by Chernoff and Zacks [6], who estimated the mean of a normal distribution for each segment in a Bayesian framework. Smith [7] proposed a Bayesian change-point model for finite series with normal and binomial models. To detect change-points in multivariate time series, Harlé *et al*. [8] used a Bayesian approach where change-points are modeled using Bernoulli variables for the change-point indicator vector. Successive generalizations and extensions of Bayesian methods for change-point problems include [9–13] and many others.

Different authors propose diverse models for each segment. For example, Punskaya *et al*. [14] suggested a Bayesian method for fitting piecewise linear regression models such as autoregressive models. To explain regime switching behaviour in the conditional mean, Chan and Tong [15] proposed a new class of non-linear models, called the Smooth Transition Autoregressive (STAR) models. This model is an extension of the Threshold Autoregressive (TAR) model, introduced by Tong and Lim [16–18]. The TAR model is a piecewise linear model consisting of two or more linear sub-models. An indicator variable is used in the TAR model which represents a switch from one regime to another and takes a value zero or one, depending upon the values of a transition variable and a threshold parameter. This indicator variable implies abrupt jumps from one regime to the next. Chan and Tong [15] suggested the replacement of the indicator function with a smooth transition function in their STAR model, since sudden jumps from one regime to another may not be the best representation of the underlying mechanism generating observed data. Davis *et al*. [19] estimated structural breaks in a nonstationary time series by segmenting the series into blocks of distinct autoregressive (AR) processes. They assumed the number of breakpoints, their locations and the orders of the respective AR models are unknown. To segment non-stationary time series data, Wood *et al*. [20] developed a Bayesian mixture of autoregressive models where components of the model are time series and mixture probabilities. The time series have constant but unknown parameters and those mixture probabilities depend on time. They assumed unknown lag of the AR processes as well as an unknown number of components in the mixture model. To estimate the number and locations of multiple change-points in the mean of a Gaussian AR(1) process, Chakar *et al*. [21] proposed a new approach where the unknown autocorrelation coefficient and the variance of an “innovation” term remain unchanged from one segment to the other. They firstly estimated the autocorrelation coefficient and then decorrelate the series. After that they applied a dynamic programming algorithm to the decorrelated series.

As noted above, some existing models for time series segmentation have used a segmented AR model [19, 22]. This paper proposes to segment time series data using a segmented ARMA model, an approach that is surprisingly absent from the existing literature. Results for the well-log time series data discussed below show that fitting an ARMA model in each segment potentially identifies a greater number of change-points than the AR model in both real and simulated data and thus provides higher sensitivity. Moreover, we find that this is true for both large and small values of the variance *σ*^2^ of the innovation term. Detailed explanations and comparison with AR model are provided in S3 Appendix.

The Bayesian segmented ARMA change-point model presented here resembles the approach of Keith *et al*. [23]. The major modelling innovation here is the use of an ARMA model in all segments. The posterior marginal distribution of this model are difficult to analyse directly since they have nonstandard form. However, simulated sampling can be performed via a Markov chain Monte Carlo (MCMC) algorithm. Several MCMC algorithms are available in the literature including the Metropolis-Hastings algorithm [24, 25], Gibbs Sampler [26], the Reversible Jump MCMC algorithm [9]; Multiple-Try Metropolis algorithm [27] and Delayed Rejection Metropolis-Hastings algorithm [28]. The Bayesian segmented ARMA change-point model here uses a highly efficient sampling technique known as the Generalized Gibbs Sampler (GGS) for generating samples from a posterior distribution [29]. The dimension in this algorithm does not need to be fixed and it provides flexibility to sample from varying dimensional spaces. The Generalized Gibbs Sampler (GGS) has been applied to some very high dimensional problems (see [30, 31]). It has resulted in highly efficient sampling for these problems. However, no systematic comparison of the advantages and disadvantages of the GGS versus the reversible jump sampler has been performed.

## Methodology

### Problem statement

We consider the problem of modeling a time series by segmenting the series into blocks of autoregressive moving average (ARMA) processes. Let *t* = 1, ⋯, *T* be time points in the signal or time series, where *T* represents the total length of the signal. Let *x*_*t*_ represent a real valued signal at time point, *t*. Let, $X={\left(x_{t}\right)}_{t=1}^{T}$ represent the time series vector or the signal that we want to segment. The ARMA model is:

$$\begin{array}{c} x_{t}=c+\epsilon_{t}+\sum_{i=1}^{a}\psi_{i}\left(x_{t-i}-c\right)+\sum_{i=1}^{m}\theta_{i}\epsilon_{t-i}. \end{array}$$

where *ψ*_1_, ⋯, *ψ*_*a*_ and *θ*_1_, ⋯, *θ*_*m*_ are the parameters of the AR and MA sub-models, respectively; *a* and *m* denote the order of the AR and MA sub-models; *c* is the mean of the ARMA model; *ϵ* is white noise and *x*_*t*_ is the time series. The number of change-points and their locations are assumed unknown. Each segment in the time-series is assumed to be generated by an ARMA model with different mean, and the goal is to infer the most probable segment locations and model parameters that describe them.

### Likelihood model

We start by writing down the likelihood function of a model in which the sequence within each segment is generated by an ARMA process. This determines the probability of generating the observed sequence for any given parameter values. For each position in the signal except the first, the probability of starting a new segment at that position is denoted by *ϕ*. Thus a time series with *K* segments that have starting positions **s** = (1 = *s*_1_ < ⋯ < *s*_*K*_ ≤ *T*) is generated with probability:

$$\begin{array}{c} p\left(K,s|\phi \right)=\phi^{K-1}{\left(1-\phi \right)}^{T-K}. \end{array}$$ (1)

Here, *s*_1_ = 1, indicating that the first segment always starts at the beginning of the signal. Let the right hand points of the segments be **d** = (*d*_1_, ⋯, *d*_*K*_) where *d*_*K*_ = *T* so that the last segment always finishes at the end of the signal. Let *X*_*k*_ be the signal of the segment between positions *s*_*k*_ and *d*_*k*_ inclusive. Each segment is then assigned to one of *N* groups with probabilities ***π*** = (*π*_1_, ⋯, *π*_*N*_) where *π*_*n*_ is the probability of assigning any segment to group *n*. We denote the group to which segment *k* is assigned by *g*_*k*_ ∈ {1, ⋯, *N*} where **g** = (*g*_1_, ⋯, *g*_*K*_). Then the probability of a specific assignment of the *K* segments to the *N* groups is

$$\begin{array}{c} p\left(g|K,\pi \right)=\prod_{k=1}^{K}\pi_{g_{k}}. \end{array}$$ (2)

Let *b*_*n*_ be the number of segments with *g*_*k*_ = *n*. The probability of a specific assignment of the *K* segments to the *N* groups can be alternatively defined as:

$$\begin{array}{c} p\left(g|K,\pi \right)=\prod_{n=1}^{N}\pi_{n}^{b_{n}}. \end{array}$$ (3)

Each segment is then modeled by an ARMA model. We write the ARMA model in each segment as:

$$\begin{array}{c} x_{t}=c_{k}+\epsilon_{t}+\sum_{i=1}^{a}\psi_{i}\left(x_{t-i}-c_{k}\right)+\sum_{i=1}^{m}\theta_{i}\epsilon_{t-i}. \end{array}$$ (4)

Here, *c*_*k*_ is the mean signal level for segment *k* and $c_{k}∼N\left(\mu_{g_{k}},\tau_{g_{k}}^{2}\right)$, where $\mu_{g_{k}}$ and $\tau_{g_{k}}^{2}$ are the mean and variance of the distribution of these means for the group *g*_*k*_. Also, ***ϵ*** = (*ϵ*_1_, ⋯, *ϵ*_*T*_) is the vector of error terms and *ϵ*_*t*_ = *x*_*t*_ − λ_*t*_, where $\lambda_{t}=c_{k}+\sum_{i=1}^{a}\psi_{i}\left(x_{t-i}-c_{k}\right)+\sum_{i=1}^{m}\theta_{i}\epsilon_{t-i}$. We suppose that $\epsilon_{t}∼N\left(0,\sigma^{2}\right)$, where *σ*^2^ is the variance of error terms and this applies for *t* ∈ (*s*_*k*_, ⋯, *d*_*k*_). In our model only these segment means differ between segments; other parameters of the ARMA model, ***ψ*** and ***θ***, are the same for all segments. This assumption is appropriate for the applications used in this paper but other data sets may need to allow all of these parameters to differ between segments. In this paper, when we define the order of the AR (*a*) and MA (*m*) submodels we only intend for *a* and *m* to take values 0 or 1. Note that when *t* − *s*_*k*_ is less than *a* or *m*, the expression for λ_*t*_ includes terms from the previous segment. However, for the left end of the signal when we can not look back *a* or *m* order steps, λ_*t*_ includes only the *c*_*k*_ term. An alternative is to choose the initial values for each segment by initializing them from the stationary distribution for the ARMA process in that segment.

The probability density of the observed signal is a product over all segments, that is, the probability of the signal **X** conditioning on parameters *K*, **s**, ***θ***, ***ψ***, **c** and *σ*^2^ is expressed as a product of normal distributions with mean λ_*t*_ and variance *σ*^2^ as follows:

$$\begin{array}{c} \begin{array}{cc} p(X|K,s,\theta ,\psi ,c,\sigma^{2}) & =\prod_{t=1}^{T}p\left(x_{t}|K,s,\theta ,\psi ,c,\sigma^{2},x_{<t}\right)\\ & =\prod_{t=1}^{T}N\left(x_{t}|\lambda_{t},\sigma^{2}\right). \end{array} \end{array}$$ (5)

Here, *x*_<*t*_ indicates the signal value at time points 1, 2, ⋯, *t* − 1. The joint distribution of **X**, *K*, **s**, **g** and **c** conditional on the other parameters is given by:

$$\begin{array}{c} \begin{array}{cc} p(X,K,s,g,c|\phi ,\pi ,\theta ,\psi ,\sigma^{2},\mu ,\tau ) & =\\ p(X|K,s,\theta ,\psi ,c,\sigma^{2})\times \\ p(K,s|\phi )\times \\ p(c|g,\mu ,\tau )\times \\ p(g|K,\pi ) \end{array} \end{array}$$ (6)

Here, *p*(**c**|**g**, ***μ***, ***τ***) is the probability of the ARMA mean for all segments given by:

$$\begin{array}{c} p\left(c|g,\mu ,\tau \right)=\prod_{k=1}^{K}N\left(c_{k}|\mu_{g_{k}},\tau_{g_{k}}^{2}\right). \end{array}$$ (7)

**Fig 1:**
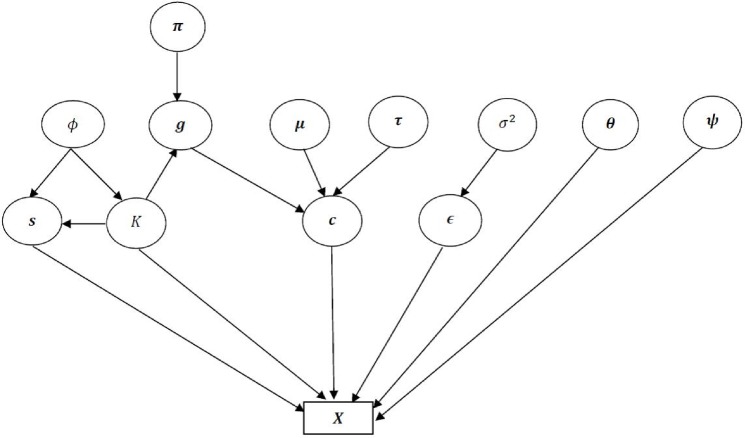
The conditional dependencies of the parameter.

Fig 1 shows the parameters of this model and their conditional dependencies. A parameter at the head of an arrow is conditionally dependent on the parameter at the tail.

### Prior distribution

Since the segmented ARMA model is presented here in a hierarchical Bayesian framework, we have to assign prior distributions for the unspecified parameters. A beta prior is assigned for *ϕ* with parameters *a* = 1.0 and *b* = 1.0. Our computational algorithm is not sensitive to the choice of prior for *ϕ*, as we show in S4 Appendix. We assign a Dirichlet distribution to ***π*** = (*π*_1_, ⋯, *π*_*N*_) with parameters (*α*_1_, ⋯, *α*_*N*_) = (1.0, ⋯, 1.0) and $\sum_{n=1}^{N}\pi_{N}=1$. Inferences are performed here using a weakly informative normal prior with mean 0.0 and variance 1.0 for the mean *μ*_*n*_ of the distribution of ARMA means in segment class *N*. We also assign an inverse gamma prior distribution with parameters *α* = 3.0 and *β* = 3.0 for the variance $\left(\tau_{n}^{2}\right)$ of the distribution of **c** and the variance of the error terms (*σ*^2^) of the ARMA model. The order of the AR model and the MA model will be considered fixed. Since we have no strong prior beliefs about the parameters of the ARMA model, we choose a uniform prior distribution for these on the interval (-1,1) and assume they are independent of each other. The forms of these hyper-priors were chosen to reflect the degree of prior belief about their respective parameters. Note that in general the signal *x*_*t*_ can be shifted and scaled so that the above prior is appropriate.

### Posterior distribution

Using Bayes’ theorem the posterior distribution of parameters is:

$$\begin{array}{c} p(K,s,g,c,\phi ,\pi ,\theta ,\psi ,\sigma^{2},\mu ,\tau \left|X\right)\\ \propto p\left(X,K,s,g,c|\phi ,\pi ,\theta ,\psi ,\sigma^{2},\mu ,\tau \right)p\left(\phi \right)p\left(\pi \right)p\left(\theta \right)p\left(\psi \right)p\left(\sigma^{2}\right)p\left(\mu \right)p\left(\tau \right) \end{array}$$ (8)

See S1 Appendix for details of the calculation of the conditional posterior distribution of each parameter.

### Sampling

**Fig 2:**
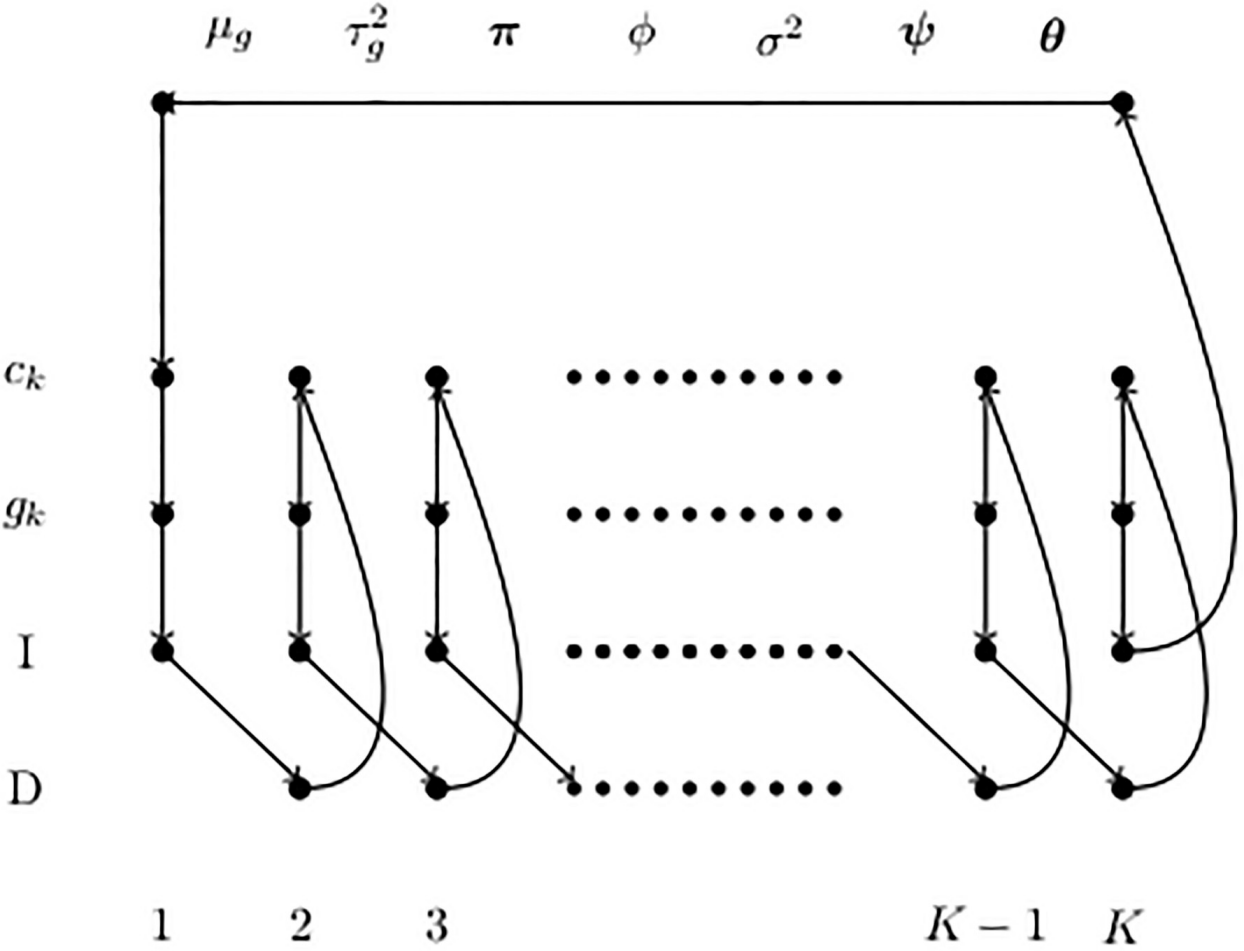
Order of move-types for the sampler. Note that *I* updates run from 1 to *K* whereas *D* updates run from 2 to *K*.

The posterior distribution obtained in S1 Appendix is sampled using a Markov chain Monte Carlo technique known as the Generalized Gibbs Sampler, or GGS [29]. The GGS algorithm cycles through a sequence of steps in which parts of a sampled element are updated, while other parts are held constant. These different types of update are analogous to the coordinate updates of the conventional Gibbs sampler and are known as “move-types”. This technique resembles a conventional Gibbs sampler but can be applied in a transdimensional setting, where it provides an alternative to the reversible jump sampler. This technique allows the number of change-points to vary: it cycles through segments inserting and deleting change-points, and shifting change-point positions. See S2 Appendix for details of the GGS algorithm. The three main stages of this algorithm in Bayesian change-point segmented ARMA model are: (also see Fig 2)

- Iterate through the segments doing insertion and deletion updates, segment group assignments (*g*_*k*_) and segment mean updates (*c*_*k*_).
- Iterate through the groups updating group parameters (group mean *μ*_*g*_ and group variance $\tau_{g}^{2}$).
- Update all the other parameters (***π***, *σ*^2^, *ϕ*, ***θ*** and ***ψ***).

#### Move types

Fig 2 illustrates the following defined move-types:

- (*I*, *k*): Decide whether to insert a new change-point in segment *k*, and at what position.
- (*D*, *k*): Decide whether to remove change-point *k* or move it to a new position (for each change-point except the first).
- *c*_*k*_: Update mean signal level *c*_*k*_ in segment *k* = 1, 2, 3, ⋯, *K*.
- *g*_*k*_: Update segment group assignments *g*_*k*_ in segment *k* = 1, 2, 3, ⋯, *K*.
- *μ*_*g*_: Update group mean *μ*_*g*_ for group *g*.
- $\tau_{g}^{2}$: Update group variance $\tau_{g}^{2}$ for group *g*.
- (***θ***, ***ψ***): Update ***θ*** and ***ψ***.
- (***π***, *σ*^2^, *ϕ*): Update all other parameters, ***π***, *σ*^2^ and *ϕ*.

There are *K* I-moves, *K* − 1 D-moves, *K* moves for updating segment group assignments, *K* moves for updating mean signal level, *N* moves for updating group mean, *N* moves for updating group variance, *a* moves to update parameters of the AR model, *m* moves to update parameters of the MA model and finally three moves for updating other parameters ***π***, *σ*^2^ and *ϕ*. The total number of moves for a sequence with *K* segments is:

$$\begin{array}{c} T(K)=4K-1+2N+a+m+3. \end{array}$$ (9)

where *N* is the number of groups, *a* is the order of the AR model and *m* is the order of the MA model.

#### Insertion: Step (*I*, *k*)

Each move-type of a GGS sampler involves drawing from a distribution over a subset of the target space. The insertion and deletion move-types mentioned in Fig 2 both involve selecting an element from a set containing only two elements. In this respect, these move-types resemble a Metropolis-Hastings update, in which a new element is proposed and either accepted or rejected with some probability. In fact the insertion and deletion move-types involve the same subsets: they differ only in which element of the set is regarded as the source or current element, and which is regarded as the proposed element. The two elements in these subsets each have non-zero probability of being selected, proportional to the target distribution multiplied by the probability of selecting the move type when that element is the current element (see [29] for details).

In an (*I*, *k*) insertion step (where *k* ∈ {1, ⋯, *K*}) the subset contains the current segmentation and a new segmentation with a change-point inserted somewhere in segment *k*. A new segment end-point position *z* is proposed between *s*_*k*_ and *d*_*k*_ − 1, inclusive. The location of *z* is selected from a uniform distribution over the set {*s*_*k*_, ⋯, *d*_*k*_ − 1}. Then for the left segment (from *s*_*k*_ to *z*) new values of $g_{k}^{\prime }$ and $c_{k}^{\prime }$ are proposed and for the right segment (from *z* + 1 to *d*_*k*_) new values of $g_{k+1}^{\prime \prime }$ and $c_{k+1}^{\prime \prime }$ are proposed. Here, $g_{k}^{\prime }$ and $g_{k+1}^{\prime \prime }$ are selected from a discrete distribution with parameters *π*_1_, …, *π*_*N*_ and then $c_{k}^{\prime }$ and $c_{k+1}^{\prime \prime }$ are selected from normal distributions with parameters $\left(\mu_{g_{k}^{\prime }},\tau_{g_{k}^{\prime }}^{2}\right)$ and $\left(\mu_{g_{k+1}^{\prime \prime }},\tau_{g_{k+1}^{\prime \prime }}^{2}\right)$ respectively. Choosing $g_{k}^{\prime }$, $g_{k+1}^{\prime \prime }$, $c_{k}^{\prime }$ and $c_{k+1}^{\prime \prime }$ in this manner results in cancellations such that the terms *p*(**c**|**g**, ***μ***, ***τ***) and *p*(**g**|*K*, ***π***) in Eq 6 disappear when calculating the acceptance ratio.

After further cancellations, the new change-point at position *z* + 1 is rejected with a probability proportional to

$$\begin{array}{c} P_{1}=\left(1-\phi \right)\prod_{t=s_{k}}^{d_{k}}p\left(\epsilon_{t}|0,\sigma^{2}\right)\frac{1}{\left(d_{k}-s_{k}\right)}\frac{1}{T(K)} \end{array}$$ (10)

where

$$\begin{array}{c} p\left(\epsilon_{t}|0,\sigma^{2}\right)=\frac{1}{\sqrt{2\pi \sigma^{2}}}\;\text{exp}[-\frac{\epsilon_{t}^{2}}{2\sigma^{2}}] \end{array}$$

and

$$\begin{array}{c} \epsilon_{t}=x_{t}-\lambda_{t}=x_{t}-(c_{k}+\sum_{i=1}^{a}\psi_{i}\left(x_{t-i}-c_{k}\right)+\sum_{i=1}^{m}\theta_{i}\epsilon_{t-i}). \end{array}$$

In this expression, 1/(*d*_*k*_ − *s*_*k*_) is the probability of proposing the location of the new change-point and 1/*T*(*K*) is a correction factor used by the GGS to account for the number of move types available for the current segmentation with *K* segments.

Alternatively, the new change-point is accepted with probability proportional to:

$$\begin{array}{c} P_{0}=\phi \prod_{t=s_{k}}^{z}p\left(\epsilon_{t}^{\prime }|0,\sigma^{2}\right)\times \prod_{t=z+1}^{d_{k}}p\left(\epsilon_{t}^{\prime \prime }|0,\sigma^{2}\right)\frac{1}{T(K+1)} \end{array}$$ (11)

where

$$\begin{array}{c} p(\epsilon_{t}^{\prime }|0,\sigma^{2})=\frac{1}{\sqrt{2\pi \sigma^{2}}}\;\text{exp}[-\frac{\epsilon_{t}^{\prime 2}}{2\sigma^{2}}]\\ p(\epsilon_{t}^{\prime \prime }|0,\sigma^{2})=\frac{1}{\sqrt{2\pi \sigma^{2}}}\;\text{exp}[-\frac{\epsilon_{t}^{\prime \prime 2}}{2\sigma^{2}}]\\ \epsilon_{t}^{\prime }=x_{t}-\lambda_{t}^{\prime }=x_{t}-(c_{k}^{\prime }+\sum_{i=1}^{a}\psi_{i}\left(x_{t-i}-c_{k}^{\prime }\right)+\sum_{i=1}^{m}\theta_{i}\epsilon_{t-i}^{\prime }) \end{array}$$ (12)

and

$$\begin{array}{c} \epsilon_{t}^{\prime \prime }=x_{t}-\lambda_{t}^{\prime \prime }=x_{t}-(c_{k}^{\prime \prime }+\sum_{i=1}^{a}\psi_{i}\left(x_{t-i}-c_{k}^{\prime \prime }\right)+\sum_{i=1}^{m}\theta_{i}\epsilon_{t-i}^{\prime \prime }) \end{array}$$ (13)

Here, Eq 12 applies for the left segment from *s*_*k*_ to *z* and Eq 13 applies for the right segment from *z* + 1 to *d*_*k*_.

Thus the new change-point at *z* + 1 is accepted with probability $\frac{P_{0}}{P_{0}+P_{1}}$ or rejected with probability $\frac{P_{1}}{P_{0}+P_{1}}$. An alternative (which we have not implemented) is to use the Metropolis-Hastings acceptance probability min{1, *P*_0_/*P*_1_}.

If a change-point is inserted, the move-type is updated to (*D*, *k*+ 1), otherwise it remains (*I*, *k*). In either case, the move-type is then further updated as in Fig 2.

#### Deletion: Step (*D*, *k*)

For each segment *k* = 2, ⋯, *K*, a (*D*, *k*) deletion step also involves selecting an element from a set containing only two elements: the current segmentation and a new segmentation with the change-point at the left end of segment *k* removed, so that segments *k* − 1 and *k* merge to form a new segment. Values of *g*_*k*_ and *c*_*k*_ are then chosen for the new merged segment: *g*_*k*_ is selected from a discrete distribution with parameters *π*_1_, …, *π*_*N*_ and then *c*_*k*_ is selected from a normal distribution with parameters $\mu_{g_{k}}$ and $\tau_{g_{k}}^{2}$ respectively. Other parameters and the positions of other change-points are held constant.

The probability of accepting the deletion is proportional to:

$$\begin{array}{c} P_{1}=\left(1-\phi \right)\prod_{t=s_{k-1}}^{d_{k}}p\left(\epsilon_{t}|0,\sigma^{2}\right)\frac{1}{\left(d_{k}-s_{k-1}\right)}\frac{1}{T(K-1)} \end{array}$$

where

$$\begin{array}{c} p\left(\epsilon_{t}|0,\sigma^{2}\right)=\frac{1}{\sqrt{2\pi \sigma^{2}}}\;\text{exp}[-\frac{\epsilon_{t}^{2}}{2\sigma^{2}}] \end{array}$$

and

$$\begin{array}{c} \epsilon_{t}=x_{t}-\lambda_{t}=x_{t}-(c_{k}+\sum_{i=1}^{a}\psi_{i}\left(x_{t-i}-c_{k}\right)+\sum_{i=1}^{m}\theta_{i}\epsilon_{t-i}) \end{array}$$

Here *K* is the number of segments in the current segmentation, that is, without making the deletion.

The probability of rejecting the deletion is proportional to:

$$\begin{array}{c} P_{0}=\phi \prod_{t=s_{k-1}}^{z}p\left(\epsilon_{t}^{\prime }|0,\sigma^{2}\right)\times \prod_{t=z+1}^{d_{k}}p\left(\epsilon_{t}^{\prime \prime }|0,\sigma^{2}\right)\frac{1}{T(K)}. \end{array}$$

Thus the deletion is accepted with probability $\frac{P_{1}}{P_{0}+P_{1}}$ or rejected with probability $\frac{P_{0}}{P_{0}+P_{1}}$.

The conditional posterior distribution for **c** and ***ϵ*** is proportional to:

$$\begin{array}{c} \prod_{t=s_{k}}^{d_{k}}p\left(x_{t}|\lambda_{t},\sigma^{2}\right)\times p\left(c_{k}|\mu_{g_{k}},\tau_{g_{k}}^{2}\right). \end{array}$$

The **c**’s and the corresponding $\epsilon_{s_{k}},\cdots ,\epsilon_{s_{k+1}}$ can be updated one segment at a time with this conditional posterior distribution. Now, the conditional posterior distribution for ***θ*** and ***ϵ*** is proportional to:

$$\begin{array}{c} \prod_{t=1}^{T}p\left(x_{t}|\lambda_{t},\sigma^{2}\right)\times p\left(\theta \right). \end{array}$$

To update ***ψ***, similar procedures were used. The sampler iterates through the groups, updating group parameters (***μ*** and ***τ***) with their respective conditional posterior distributions.

The sampler uses conventional Gibbs updates for other parameters *c*_*k*_, *g*_*k*_, *θ*_*i*_, *ϕ*_*i*_, *σ*^2^, ***π***, *ϕ*, *μ*_*g*_ and *τ*_*g*_. We use the Slice sampler [32] where needed to draw from non-standard distributions.

## Validation of the methodology

We have used a simulation-based method for testing the correctness of software for fitting Bayesian models using posterior simulation [33]. This validation technique is based on posterior quantiles. Consider the general Bayesian joint distribution *p*(*y*|Θ)*p*(Θ), where *p*(*y*|Θ) presents the sampling distribution of the data, *p*(Θ) presents the proper prior distribution of the parameter vector Θ, and inferences are based on the posterior distribution, *p*(Θ|*y*). The validation method samples a parameter vector Θ^(0)^ from *p*(Θ). Then conditional on Θ^(0)^, this technique samples data *y* from *p*(*y*|Θ = Θ^(0)^) and then simulates sampling from the posterior distribution, *p*(Θ|*y*) using the software to be validated. The resulting posterior sample of size *L* is denoted (Θ^(1)^, Θ^(2)^, ⋯, Θ^(*L*)^). Finally, for each coordinate of Θ^(0)^, denoted *θ*^(0)^, compute its posterior quantile $\hat{q}\left(\theta^{\left(0\right)}\right)$, with respect to the posterior sample (*θ*^(1)^, *θ*^(2)^, ⋯, *θ*^(*L*)^). To perform the validation procedure, many replications are required, each drawing Θ^(0)^ from *p*(Θ) and *y* from *p*(*y*|Θ^(0)^). The simulation output is a collection of estimated posterior quantiles. From these quantiles calculate a test statistic $X_{\theta }^{2}=\sum_{i=1}^{N_{rep}}{\left(\Phi^{-1}\left(q_{i}\right)\right)}^{2}$ where, $q_{i}=\frac{1}{L}\sum_{l=1}^{L}I_{\theta_{i}^{\left(0\right)}>\theta_{i}^{\left(l\right)}}$ is the posterior quantile for the ith replication, *N*_*rep*_ is the total number of replications, *θ* denotes component of Θ and Φ represents the standard normal CDF. If the software is implemented correctly, this test statistic follows a *χ*^2^ distribution with *N*_*rep*_ degrees of freedom and also the posterior quantiles will be uniformly distributed. The posterior quantiles’ deviation from uniformity can be quantified by calculating the associated *p* value, that is, *p*_*θ*_ for each $X_{\theta }^{2}$. Extremely small *p*_*θ*_ values indicate an error in the software. As an exploratory tool, *p*_*θ*_ values can be transformed into a *z*_*θ*_ statistic (*z*_*θ*_ = Φ^−1^(*p*_*θ*_)). If all |*z*_*θ*_| statistics are not extreme, such as less than 2, the software may be considered validated.

To validate our method according to the above described technique, we generated the parameters *τ*^2^, *σ*^2^, *π*, *μ* from the following prior distributions:

$$\begin{array}{c} \tau^{2}∼Inv-gamma\left(3,3\right)\\ \sigma^{2}∼Inv-gamma\left(3,3\right)\\ \pi ∼U(0,1)\\ \mu ∼N(0,1)\\ \theta ∼U(-1,1)\\ \psi ∼U(-1,1) \end{array}$$

**Fig 3:**
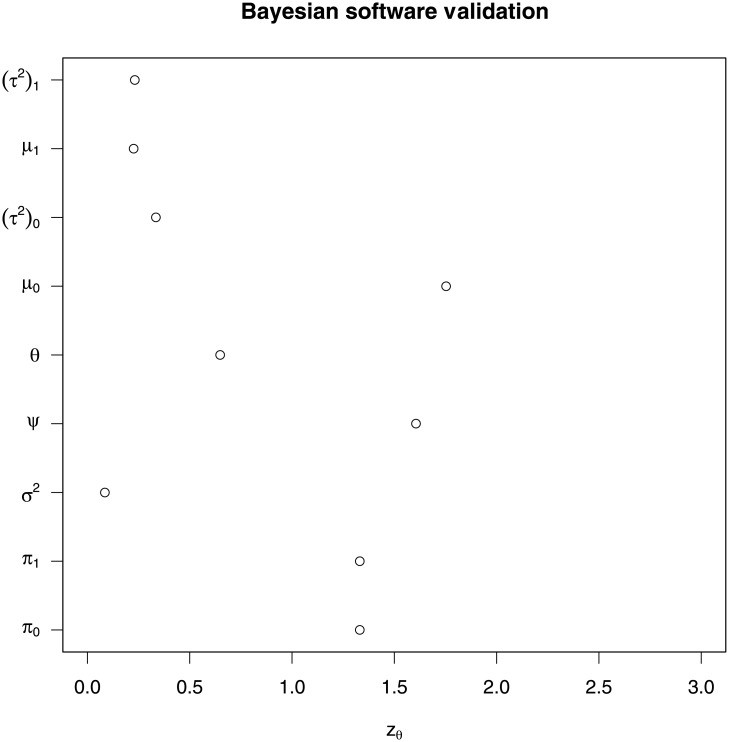
Absolute *z*_*θ*_ statistic plot. Each row shows a parameter of the segmented ARMA model and the |*z*_*θ*_| statistics associated with these parameters are displayed as a circle in each row.

We generated 20 sequences from an ARMA model with 20 different segment means where every sequence has length 100. We simulated 5000 draws from the posterior distribution of the model parameters. Then the quantiles of the posterior distributions for each parameter were determined and the whole procedure was repeated 20 times. From these quantiles we determined the absolute values of the *z*_*θ*_ statistic. The absolute *z*_*θ*_ statistics from this simulation are plotted in Fig 3:

In the above plot, since the *z*_*θ*_ statistic for each parameter is less than 2, we conclude the software is correctly written. More precisely, we find no evidence of software errors.

## Illustrative examples

### Simulation example

**Fig 4:**
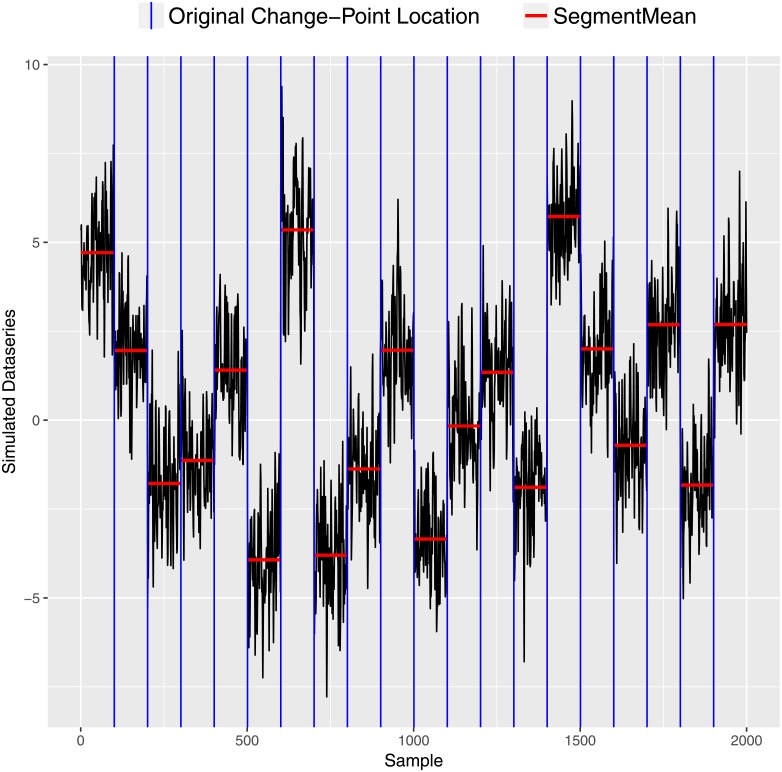
Simulated signal. The true change-point locations are shown as vertical blue lines and segment means are shown as horizontal red lines.

As a test of our method, we applied it to a simulated example in which the number and location of change-points, ARMA parameters, segment means and error variance are known. We analyzed 20 time series, each containing 100 observations, generated from the autoregressive moving average (ARMA(1,1)) model with parameter values *ψ* = 0.22 and *θ* = 0.60. Each series was generated using *σ*^2^ = 0.96 and 20 different segment means. The simulated ARMA data with the true segment means and the location of change-points is shown in Fig 4.

**Fig 5:**
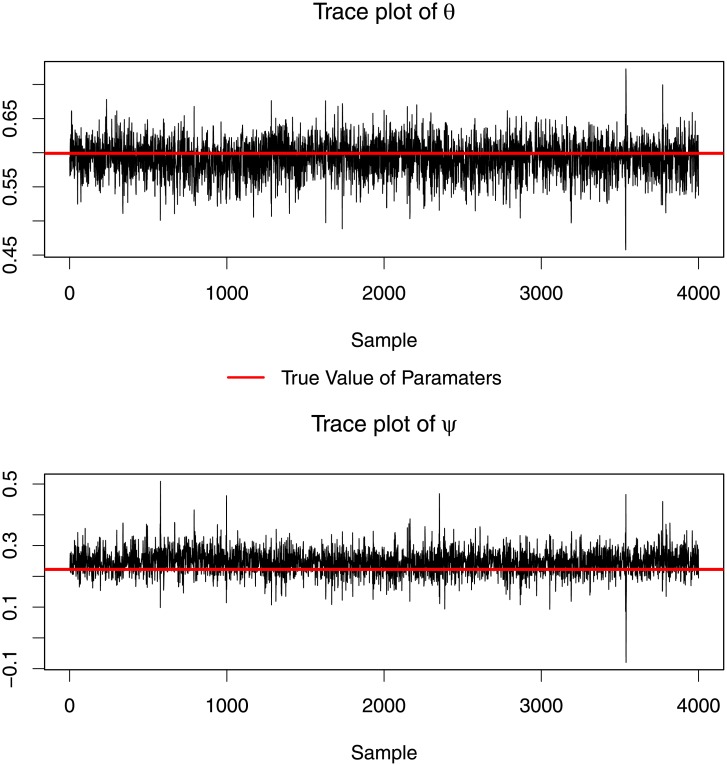
Trace plot of AR and MA parameters. Both parameters converged to the true values.

We executed 5,000 iterations of the MCMC estimation algorithm and the first 1,000 iterations were treated as a burn in period and discarded. The convergence of AR and MA parameters is evident in Fig 5. Both AR and MA parameters converge, display good mixing and are close to the true values of those parameters.

**Fig 6:**
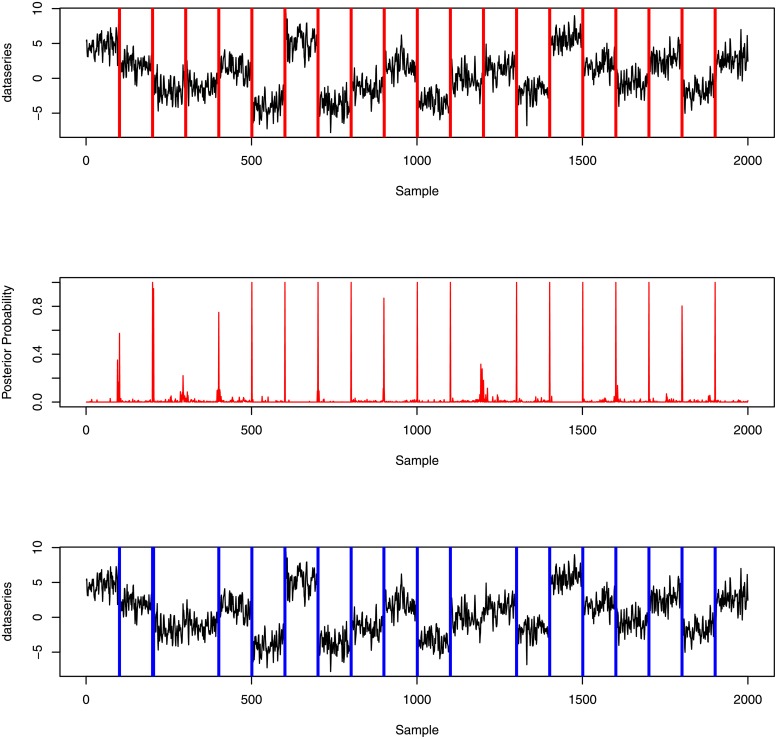
(Top) Segmented signal with the true change-point locations. (Middle) Posterior probabilities of occurrence of change-points. (Bottom) Estimated change-point locations (posterior probability greater than 0.5). The middle plot shows the location of peaks in the probability profile closely follows the true change-points locations but in some positions with low posterior probability. Using a threshold in posterior probability 0.5, we identify 17 change-points out of 19 which match the locations of the true change-points.

The top panel of Fig 6 presents the simulated signal with the true change-points (red vertical line). The middle plot shows the posterior distribution of occurrence of change-point locations, that is, the posterior probability of being change-points at each position. The height of the (red) ‘spikes’ indicates the posterior probability of a change-point being selected at each time point. The top two plots show the locations of estimated change-points and the true change-points are similar. This picture is clearer if we compare actual change-point locations and estimated change-point locations with the simulated data, as in the top and bottom plots of Fig 6. The blue lines indicate time points at which the posterior probabilities of change-points are greater than 0.5. Fig 6 (bottom) identifies 17 change-points out of 19 true change-points.

**Fig 7:**
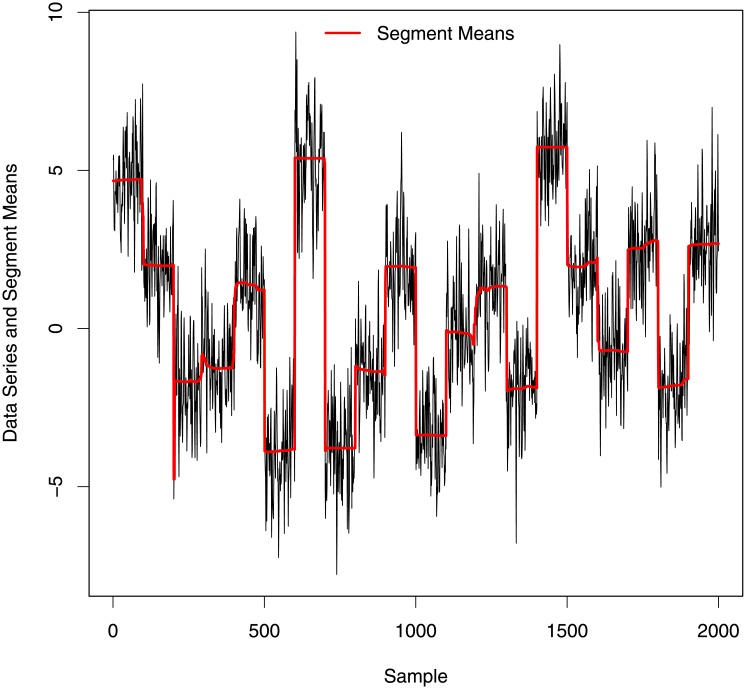
Segment means at each position of simulated data.

Fig 7 plots posterior estimators of mean signal level (*c*) at each position of the simulated signal. This plot clearly indicates 20 segments in the simulated signal detecting a change in mean even where change-points were not detected in Fig 6.

### Well-log data

**Fig 8:**
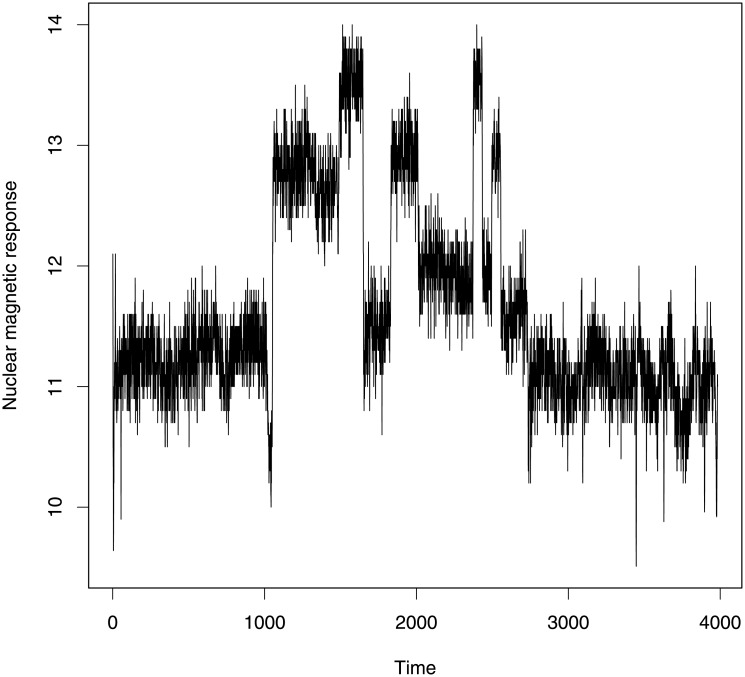
Well-log data. This data provides information about the rock structure of the well. Some change-points are present in the data reflecting the presence of a new rock.

We now apply the segmented ARMA model to identify change-points in a real data set. This data records 4050 measurements of nuclear magnetic response of underground rocks during the drilling of a well. During drilling, data were obtained at discrete time points by lowering a probe into a bore-hole in the Earth’s surface. This geophysical data originates from Ó Ruanaidh and Fitzgerald [34] and has been previously analyzed in the context of change-point detection by many researchers, for example, by Fearnhead and Clifford [35], Fearnhead [36] and by Whiteley *et al*. [1]. A few outliers present in this data were removed by hand before analyzing the data as in Fearnhead [36]. The data is shown in Fig 8.

The piecewise constant signal of this data indicates information about the geophysical structure of the rocks in the well. Changes in mean occur at each time point whenever a new rock type is found. To identify change-points in this well-log data, Ó Ruanaidh and Fitzgerald [34] used a Gibbs sampler to fit a Bayesian change-point model with a fixed number of change-points; Fearnhead and Clifford [35] used on-line Bayesian analysis of data with a hidden Markov model using particle filters; Fearnhead [36] considered an extension of the model described by Fearnhead and Clifford [35] in which they considered all the parameters of their model to be unknown and they used reversible jump MCMC to fit that model; Whiteley *et al*. [1] considered the same model used by Fearnhead [36] to analyze well-log data but here they used a block Gibbs sampler for generating samples from the posterior distribution.

**Fig 9:**
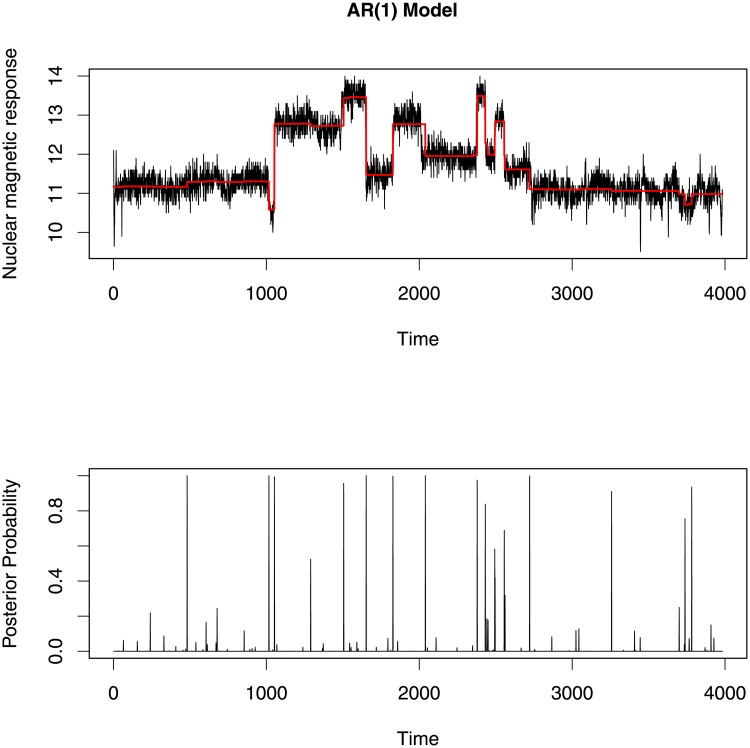
Top plot shows the posterior estimators of mean signal level (c) on each segment of AR(1) model. Bottom plot shows the posterior probability of a change-point at each position. These segment means and change-point positions indicate significant jumps in the original data.

**Fig 10:**
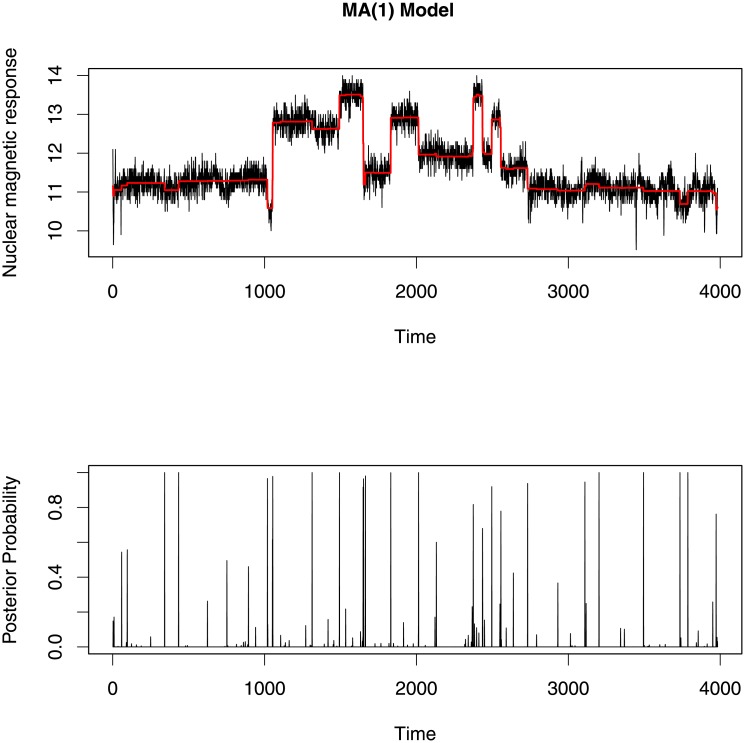
Top and bottom plot show the segment mean profiles and the change-point profiles for MA(1) model respectively. This model identifies more change-points than the AR(1) model.

**Fig 11:**
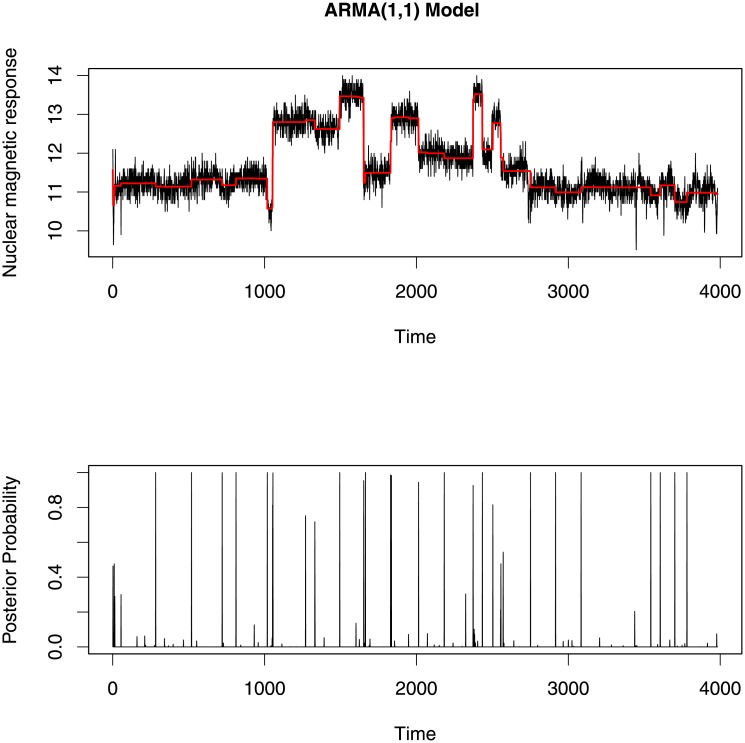
Top and bottom plot show the segment mean profiles and the change-point profiles for ARMA(1,1) model respectively. These change-points and segment means are almost identical to those identified using the MA(1) model.

To find change-points in this data, we attempted to fit an AR(1) model, an MA(1) model and an ARMA(1,1) model. We are the first to investigate this data using a segmented ARMA model. Each model was run for 5000 iterations and then tested for convergence of each parameter using trace plots and an autocorrelation function (ACF) plot which represents the degree of correlation between all pairs of samples separated by progressively larger lags (number of samples). The change-point profiles and segment mean profiles for each model are given in Figs 9–11.

In our model, the initial segmentation was generated using a probability of starting a new segment *ϕ* = 0.1. The initial segmentation was generated by throwing a uniform(0,1) random number for each sequence position except the first, making that position a change-point if the random number is less than *ϕ* at that position. Note that, this initial segmentation should not affect the stationary distribution of the Markov chain. The posterior probabilities of occurrence of change-points at each position of the input sequence are calculated using the uniform prior probability distribution for *ϕ* (the probability that any given sequence position is a change-point) and the likelihood probability (*p*(*K*, **s**|***ϕ***) = ***ϕ***^*K*−1^(1 − *ϕ*)^*T*−*K*−1^) of generating a new segmentation with *K* change-points and **s** = (1 = *s*_1_ < ⋯ < *s*_*K*_ ≤ *T*) starting positions.

All change-point profile plots (Figs 9–11) show almost the same change-points locations. But at some locations the AR(1) model gives comparatively smaller posterior probability than the MA(1) and ARMA(1,1) models. In addition, the AR(1) model identifies fewer change-points with high posterior probability (when the posterior probability is more than 0.5) than the other two models. These results are somewhat similar to the previous results found in the change-point literature [1, 34–36].

**Fig 12:**
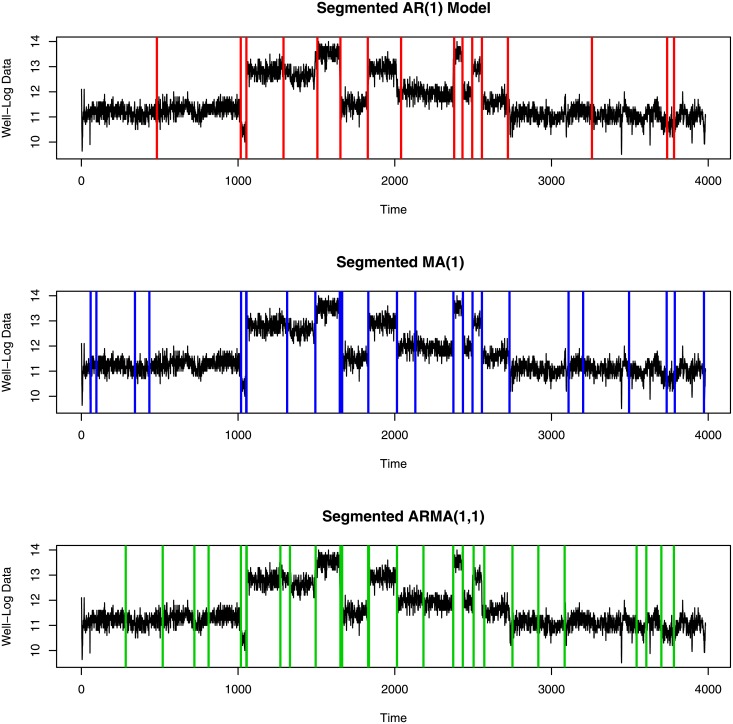
Estimated change-point locations with posterior probability greater than 0.5 for AR(1), MA(1) and ARMA(1,1) model.

Since the three segmented ARMA models indicate many of the same change-point locations, we compare the change-points locations for which posterior probabilities are greater than 0.5 in Fig 12. Among these three models, the ARMA(1,1) model identifies the largest number of change-points and matches more closely with the number and locations of change-points detectable to the eye. Moreover, the ARMA(1,1) model picks up small changes in mean with high posterior probability whereas the AR(1) and MA(1) models missed change-points at some time points where small jumps occured in the data.

To determine the best model among these three, we compare these models using the deviance information criterion (DICV). The DICV is defined as: $\text{DICV}=p_{v}+\bar{D(\theta )}$, where $\bar{D(\theta )}$ is the mean posterior deviance, *p*_*v*_ = *Var*(*D*(*θ*))/2 and deviance *D*(*θ*) = −2lnf(*y*|*θ*) (details of DICV are in [37, 38]). The DICV, $\bar{D(\theta )}$ and *p*_*v*_ of these three models are shown in Table 1.

**Table 1:** DICV of Models.

Model	$\bar{D(\theta )}$	*p*_*v*_	DICV
Segmented AR(1) Model	958.0043	181306.7	182264.7
Segmented MA(1) Model	509.5514	253045.4	253554.9
Segmented ARMA(1,1) Model	672.8849	169945.6	170618.5

Here, the segmented ARMA(1,1) model gives lower DICV than the other two models. The lower DICV of the ARMA(1,1) model supports the conclusion that the ARMA(1,1) model is the best of the three.

## Discussion

In this paper, we have developed a Bayesian change-point segmented ARMA model to segment time series data. The novel features of our approach include: (1) It uses an ARMA model in each segment (2) It uses a highly efficient sampling technique (GGS) to generate samples from a posterior distribution. Results for simulated data and real data show that this model achieves high detection accuracy.

**Fig 13:**
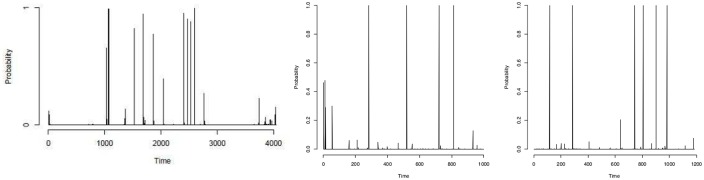
Comparison between the results of a random walk model [36] and our ARMA(1,1) model. (Left) change-point profiles of random walk model [36]; (centre) change-point profiles of ARMA(1,1) at time points 1 to 1000; (right) change-point profiles of ARMA(1,1) after time point 2800.

**Fig 14:**
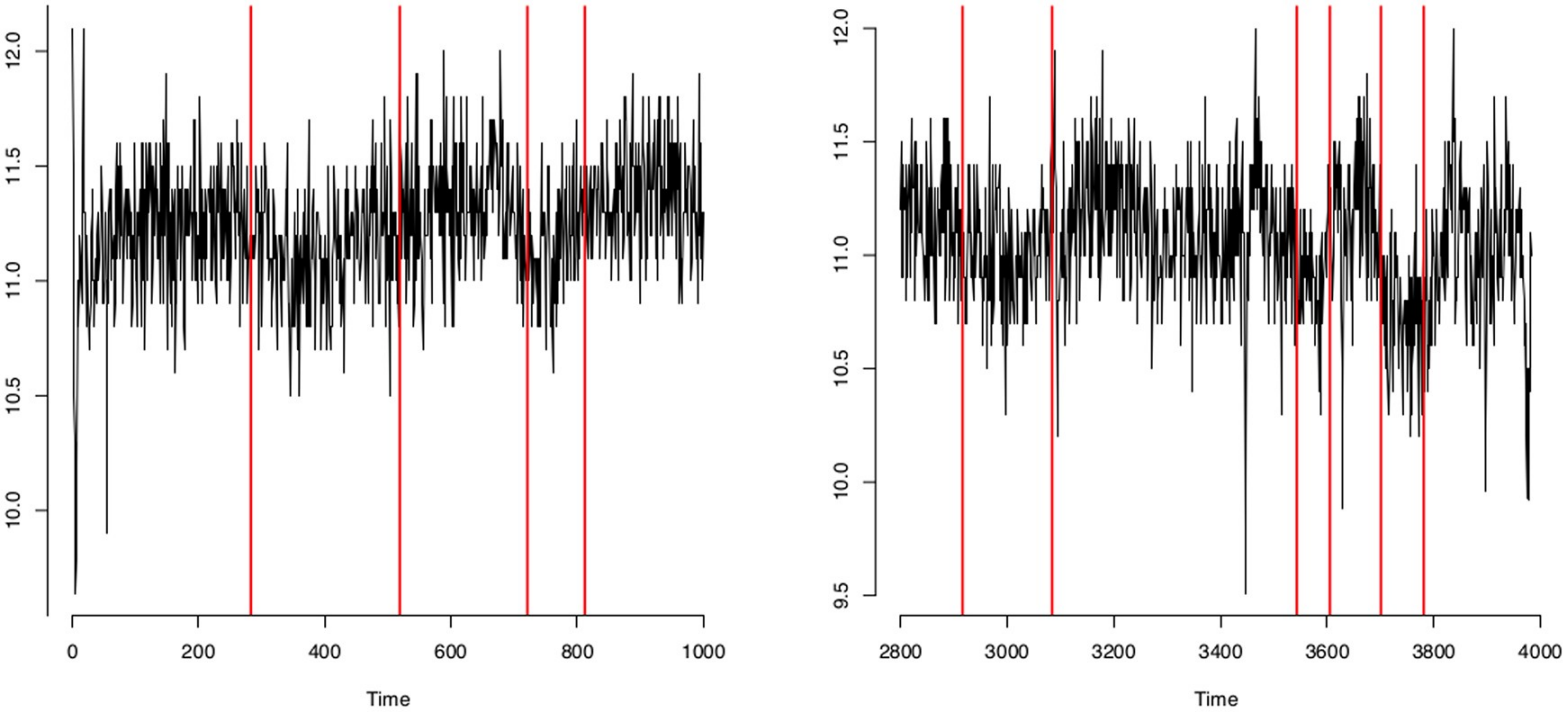
Change-point locations identified using ARMA(1,1) model displayed with the original well-log data at two different time points. (Left) change-point locations using ARMA(1,1) model in well-log data at time points 1 to 1000; (right) change-point locations using ARMA(1,1) model in well-log data after time point 2800.

The results we obtain for the well-log data seem reasonable when judged by eye. The posterior probabilities of change-point occurrences of the ARMA(1,1) model are somewhat similar to results previously found in the literature for this data. Ó Ruanaidh and Fitzgerald [34] assumed 13 change-points exist in this data (after removing outliers) but our ARMA(1,1) model identifies 27 change-points (considering posterior probability more than 0.5). They missed some small jumps in the data whereas the ARMA(1,1) model identifies small jumps as well as large jumps. Fearnhead and Clifford [35] inferred 16 change-points but since they did not remove the outliers of the data, the results cannot be directly compared. Fearnhead [36] found too many change-points in their piecewise constant model. They used a random walk sampler and found similar change-points to our ARMA(1,1) model. In addition to the change-points found by Fearnhead [36], the ARMA(1,1) model identifies some significant change-points between time points 1 and 1000 and after 2800 (shown in Figs 13 and 14).

The results of the ARMA(1,1) model are similar to the results reported in Whiteley *et al*. [1] but in some time points ARMA(1,1) model shows a higher posterior probability of change-point occurrences.

Overall, this paper has presented a promising new direction for estimation of change-point models by assuming a segment-wise ARMA model. Most of the previous methods discussed in the literature use autoregressive (AR) models in each segment. Adding a moving average component helps to consider the dependence between residual terms which is an advantage over the segmented AR model. Our results obtained using simulated data demonstrate that when the data are generated via an ARMA process, the ARMA model finds more change-points than the AR model, without finding false positives. The ARMA model also finds more change-points in the well log data, but a question remains whether the additional change-points are false positives in this case. As the true locations of change in this data set are unknown, this question cannot be answered definitively. However, we compared the segmented ARMA (1,1) model, segmented AR(1) model and segmented MA (1) model using the deviance information criterion (DICV), and found that the ARMA (1,1) is favoured by this criterion, suggesting the additional change-points reflect a real feature of the data. Since this model assumes the same variance for all segments, it is not suitable for data sets in which different segments have different variance.

## Supporting information

S1 Appendix Details of posterior distribution. (PDF)

S2 Appendix Generalized gibbs sampling. (PDF)

S3 Appendix Supplementary material A. (PDF)

S4 Appendix Supplementary material B. (PDF)
