# Supplementary material for: Bayesian change-point modeling with segmented ARMA model
Source: PLoS One. 2018 Dec 31;13(12):e0208927. doi: 10.1371/journal.pone.0208927 (PMC6312324; doi:10.1371/journal.pone.0208927)
Supplement: S1 Appendix — (PDF) [file pone.0208927.s001.pdf]

Bayesian change-point modeling with segmented ARMA model

Farhana Sadia[1], Sarah Boyd[2], Jonathan M. Keith[1*]

1 School of Mathematical Sciences, Monash University, Clayton, VIC 3800, Australia

2 Faculty of Information Technology, Monash University, Clayton, VIC 3800, Australia

* jonathan.keith@monash.edu

Supporting Information

S1 Appendix. Details of Posterior Distribution

Conditional Posterior Distribution of $\boldsymbol{\pi}$

The conditional posterior distribution of $\boldsymbol{\pi}$ is:

$$p(\boldsymbol{\pi}|\mathbf{X}, K, \mathbf{s}, \mathbf{g}, \mathbf{c}, \phi, \boldsymbol{\theta}, \boldsymbol{\psi}, \sigma^2, \boldsymbol{\mu}, \boldsymbol{\tau}) = \frac{p(\boldsymbol{\pi}, \mathbf{X}, K, \mathbf{s}, \mathbf{g}, \mathbf{c}, \phi, \boldsymbol{\theta}, \boldsymbol{\psi}, \sigma^2, \boldsymbol{\mu}, \boldsymbol{\tau})}{\int_{\boldsymbol{\pi}} p(\boldsymbol{\pi}, \mathbf{X}, K, \mathbf{s}, \mathbf{g}, \mathbf{c}, \phi, \boldsymbol{\theta}, \boldsymbol{\psi}, \sigma^2, \boldsymbol{\mu}, \boldsymbol{\tau}) d\boldsymbol{\pi}}$$

Using Eq (8), we get,

$$\frac{p(\mathbf{X}, K, \mathbf{s}, \mathbf{g}, \mathbf{c}|\phi, \boldsymbol{\pi}, \boldsymbol{\theta}, \boldsymbol{\psi}, \sigma^2, \boldsymbol{\mu}, \boldsymbol{\tau}) p(\phi) p(\boldsymbol{\pi}) p(\boldsymbol{\theta}) p(\boldsymbol{\psi}) p(\sigma^2) p(\boldsymbol{\mu}) p(\boldsymbol{\tau})}{\int_{\boldsymbol{\pi}} p(\mathbf{X}, K, \mathbf{s}, \mathbf{g}, \mathbf{c}|\phi, \boldsymbol{\pi}, \boldsymbol{\theta}, \boldsymbol{\psi}, \sigma^2, \boldsymbol{\mu}, \boldsymbol{\tau}) p(\phi) p(\boldsymbol{\pi}) p(\boldsymbol{\theta}) p(\boldsymbol{\psi}) p(\sigma^2) p(\boldsymbol{\mu}) p(\boldsymbol{\tau}) d\boldsymbol{\pi}}$$

$$= \frac{p(\mathbf{X}, K, \mathbf{s}, \mathbf{g}, \mathbf{c}|\phi, \boldsymbol{\pi}, \boldsymbol{\theta}, \boldsymbol{\psi}, \sigma^2, \boldsymbol{\mu}, \boldsymbol{\tau}) p(\boldsymbol{\pi})}{\int_{\boldsymbol{\pi}} p(\mathbf{X}, K, \mathbf{s}, \mathbf{g}, \mathbf{c}|\phi, \boldsymbol{\pi}, \boldsymbol{\theta}, \boldsymbol{\psi}, \sigma^2, \boldsymbol{\mu}, \boldsymbol{\tau}) p(\boldsymbol{\pi}) d\boldsymbol{\pi}}$$

Then, using Eq (6) and cancelling same terms from the numerator and the denominator the conditional posterior distribution of $\boldsymbol{\pi}$ becomes

$$\frac{p(\mathbf{X}|\boldsymbol{\lambda},\sigma^2)p(\mathbf{c}|\mathbf{g},\boldsymbol{\mu},\boldsymbol{\tau})p(K,\mathbf{s}|\phi)p(\mathbf{g}|K,\boldsymbol{\pi})p(\boldsymbol{\pi})}{\int_{\boldsymbol{\pi}} p(\mathbf{X}|\boldsymbol{\lambda},\sigma^2)p(\mathbf{c}|\mathbf{g},\boldsymbol{\mu},\boldsymbol{\tau})p(K,\mathbf{s}|\phi)p(\mathbf{g}|K,\boldsymbol{\pi})p(\boldsymbol{\pi})d\boldsymbol{\pi}} = \frac{p(\mathbf{g}|K,\boldsymbol{\pi})p(\boldsymbol{\pi})}{\int_{\boldsymbol{\pi}} p(\mathbf{g}|K,\boldsymbol{\pi})p(\boldsymbol{\pi})d\boldsymbol{\pi}}$$

Now, Using Eq (3), this becomes:

$$\frac{\prod_{n=1}^{N} \boldsymbol{\pi}_n^{b_n}}{\int_{\boldsymbol{\pi}} \prod_{n=1}^{N} \boldsymbol{\pi}_n^{b_n} d\boldsymbol{\pi}} = \frac{\boldsymbol{\pi}_1^{(b_1+1)-1} \times \cdots \times \boldsymbol{\pi}_1^{(b_N+1)-1}}{\int_{\boldsymbol{\pi}} \boldsymbol{\pi}_1^{(b_1+1)-1} \times \cdots \times \boldsymbol{\pi}_1^{(b_N+1)-1} d\boldsymbol{\pi}} = Dirichlet(\boldsymbol{\pi}|((b_1+1),\cdots,(b_N+1))$$

Conditional Posterior Distribution of ϕ

The conditional posterior distribution of ϕ is,

$$\frac{p(\mathbf{X}|\boldsymbol{\lambda},\sigma^2)p(\mathbf{c}|\mathbf{g},\boldsymbol{\mu},\boldsymbol{\tau})p(K,\mathbf{s}|\phi)p(\mathbf{g}|K,\phi)p(\boldsymbol{\pi})}{\int_{\phi} p(\mathbf{X}|\boldsymbol{\lambda},\sigma^2)p(\mathbf{c}|\mathbf{g},\boldsymbol{\mu},\boldsymbol{\tau})p(K,\mathbf{s}|\phi)p(\mathbf{g}|K,\boldsymbol{\pi})p(\phi)d\phi} = \frac{p(K,\mathbf{s}|\phi)p(\phi)}{\int_{\phi} p(K,\mathbf{s}|\phi)p(\phi)d\phi}$$

Using Eq (1), the conditional posterior distribution of ϕ becomes

$$\frac{\phi^{K-1}(1-\phi)^{T-K-1}}{\int_{\phi} \phi^{K-1}(1-\phi)^{T-K-1}d\phi} = Beta(\phi|K, T-K)$$

where, K = Number of segments and $T-K$ = Length of signal-number of segments.

Conditional Posterior Distribution of $\boldsymbol{\mu}$

The conditional posterior distribution of $\boldsymbol{\mu}$ is:

$$\frac{p(\mathbf{X}|\boldsymbol{\lambda},\sigma^2)p(\mathbf{c}|\mathbf{g},\boldsymbol{\mu},\boldsymbol{\tau})p(K,\mathbf{s}|\phi)p(\mathbf{g}|K,\boldsymbol{\pi})p(\boldsymbol{\mu})}{\int_{\boldsymbol{\mu}} p(\mathbf{X}|\boldsymbol{\lambda},\sigma^2)p(\mathbf{c}|\mathbf{g},\boldsymbol{\mu},\boldsymbol{\tau})p(K,\mathbf{s}|\phi)p(\mathbf{g}|K,\boldsymbol{\pi})p(\boldsymbol{\mu})d\boldsymbol{\mu}} = \frac{p(\mathbf{c}|\mathbf{g},\boldsymbol{\mu},\boldsymbol{\tau})p(\boldsymbol{\mu})}{\int_{\boldsymbol{\mu}} p(\mathbf{c}|\mathbf{g},\boldsymbol{\mu},\boldsymbol{\tau})p(\boldsymbol{\mu})d\boldsymbol{\mu}}$$

Note that, μ or μ_n will (currently) not be updated if there is less than one segment in group n. Then using Eq (7), the conditional distribution for μ_n (holding all the other μ's constant) becomes

$$\frac{\prod_{k:g_k=n} \mathcal{N}(c_k|\mu_n,\tau_n^2)}{\int_{\mu} \prod_{k:g_k=n} \mathcal{N}(c_k|\mu_n,\tau_n^2)d\mu}$$

.

Let m be the number of segments that have $g_k = n$. Then simplifying the product $\prod_{k:g_k=n} \mathcal{N}(c_k|\mu_n, \tau_n^2) = \prod_{k:g_k=n} \frac{1}{\sqrt{2\pi\tau_n^2}} exp\left[\left(\frac{(c_k-\mu_n)^2}{2\tau_n^2}\right)\right]$, the conditional posterior distribution of $\boldsymbol{\mu}$ becomes a normal distribution with mean $\frac{\sum_{k:g_k=n} c_k}{m}$ and variance $\frac{\tau_n^2}{m}$.

Conditional Posterior Distribution of σ^2

The conditional posterior distribution of σ^2 is:

$$\frac{p(\mathbf{X}|\boldsymbol{\lambda},\sigma^2)p(\mathbf{c}|\mathbf{g},\boldsymbol{\mu},\boldsymbol{\tau})p(K,\mathbf{s}|\phi)p(\mathbf{g}|K,\boldsymbol{\pi})p(\sigma^2)}{\int_{\sigma^2} p(\mathbf{X}|\boldsymbol{\lambda},\sigma^2)p(\mathbf{c}|\mathbf{g},\boldsymbol{\mu},\boldsymbol{\tau})p(K,\mathbf{s}|\phi)p(\mathbf{g}|K,\boldsymbol{\pi})p(\sigma^2)d\sigma^2} = \frac{p(\mathbf{X}|\boldsymbol{\lambda},\sigma^2)p(\sigma^2)}{\int_{\sigma^2} p(\mathbf{X}|\boldsymbol{\lambda},\sigma^2)p(\sigma^2)d\sigma^2}$$

The prior distribution for σ^2 is: $p(\sigma^2, u_0, v_0) = \frac{v_0^{u_0}}{\Gamma(u_0)}(\sigma^2)^{-u_0-1}exp\left(-\frac{v_0}{\sigma^2}\right)$. where u_0 and v_0 are the prior parameters. Now using $\boldsymbol{\epsilon} \sim \mathcal{N}(0,\sigma^2)$ and the above prior distribution and simplifying, the conditional posterior distribution of σ^2 takes the form of an inverse gamma distribution with parameter u and v, where $u = u_0 + \frac{T}{2}$ and $v = \frac{2v_0 + \sum_{t=1}^{T}\epsilon_t^2}{2} = v_0 + \frac{1}{2}\sum_{t=1}^{T}\epsilon_t^2$.

Conditional Posterior Distribution of $\boldsymbol{\tau_n^2}$

Let $\boldsymbol{\tau}$ be the vector of $\tau_1^2, \cdots \tau_N^2$, then the conditional posterior distribution of $\boldsymbol{\tau}$ is:

$$\frac{p(\mathbf{X}|\boldsymbol{\lambda},\sigma^2)p(\mathbf{c}|\mathbf{g},\boldsymbol{\mu},\boldsymbol{\tau})p(K,\mathbf{s}|\phi)p(\mathbf{g}|K,\boldsymbol{\pi})p(\boldsymbol{\tau})}{\int_{\boldsymbol{\tau}} p(\mathbf{X}|\boldsymbol{\lambda},\sigma^2)p(\mathbf{c}|\mathbf{g},\boldsymbol{\mu},\boldsymbol{\tau})p(K,\mathbf{s}|\phi)p(\mathbf{g}|K,\boldsymbol{\pi})p(\boldsymbol{\tau})d\boldsymbol{\tau}} = \frac{p(\mathbf{c}|\mathbf{g},\boldsymbol{\mu},\boldsymbol{\tau})p(\boldsymbol{\tau})}{\int_{\boldsymbol{\tau}} p(\mathbf{c}|\mathbf{g},\boldsymbol{\mu},\boldsymbol{\tau})p(\boldsymbol{\tau})d\boldsymbol{\tau}}$$

The prior distribution for $\boldsymbol{\tau}$ is: $p(\boldsymbol{\tau}, \alpha_0, \beta_0) = \prod_{n=1}^{N} \frac{\beta_0^{\alpha_0}}{\Gamma(\alpha_0)}(\tau_n^2)^{-\alpha_0-1}exp\left(-\frac{\beta_0}{\tau_n^2}\right)$. where α_0 and β_0 are the prior parameters. Note that τ_n^2 will (currently) only be updated if there is more than one segment in group n. Now using Eq (7) and the above prior distribution and simplifying, the conditional posterior distribution of $\boldsymbol{\tau}$ takes the form of an inverse gamma distribution with parameter α and β, where $\alpha = \alpha_0 + \frac{|(k:g_k=n)|}{2}$ and $\beta = \beta_0 + \frac{1}{2}\sum_{k:g_k=n}(c_k - \mu_n)^2$.

Conditional Posterior Distribution of $\mathbf{g}$

The conditional posterior distribution of $\mathbf{g}$ is:

$$\frac{p(\mathbf{X}|\boldsymbol{\lambda},\sigma^2)p(\mathbf{c}|\mathbf{g},\boldsymbol{\mu},\boldsymbol{\tau})p(K,\mathbf{s}|\phi)p(\mathbf{g}|K,\boldsymbol{\pi})p(\mathbf{g})}{\int_{\mathbf{g}} p(\mathbf{X}|\boldsymbol{\lambda},\sigma^2)p(\mathbf{c}|\mathbf{g},\boldsymbol{\mu},\boldsymbol{\tau})p(K,\mathbf{s}|\phi)p(\mathbf{g}|K,\boldsymbol{\pi})p(\mathbf{g})d\mathbf{g}} = \frac{p(\mathbf{c}|\mathbf{g},\boldsymbol{\mu},\boldsymbol{\tau})p(\mathbf{g}|K,\boldsymbol{\pi})p(g)}{\int_{\mathbf{g}} p(\mathbf{c}|\mathbf{g},\boldsymbol{\mu},\boldsymbol{\tau})p(\mathbf{g}|K,\boldsymbol{\pi})p(g)d\mathbf{g}}$$

Using Eq (2) and Eq (7), The conditional posterior distribution of $\mathbf{g}$ becomes

$$\frac{\prod_{k=1}^{K}\mathcal{N}(c_k|\mu_{g_k},\tau_{g_k}^2)\times\prod_{k=1}^{K}\pi_{g_k}}{\int_g(\prod_{k=1}^{K}\mathcal{N}(c_k|\mu_{g_k},\tau_{g_k}^2)\times\prod_{k=1}^{K}\pi_{g_k})dg}.$$

The g_k 's are independent, so can be updated one at a time with the conditional posterior distribution of g which is a discrete distribution with parameter $\frac{\mathcal{N}(c_k|\mu_{g_k},\tau_{g_k}^2)\times\pi_{g_k}}{\int_g(\mathcal{N}(c_k|\mu_{g_k},\tau_{g_k}^2)\times\pi_{g_k})dg}$.

Conditional Posterior Distribution of $\mathbf{c}$ and $\boldsymbol{\epsilon}$

The conditional posterior distribution of $\mathbf{c}$ and $\boldsymbol{\epsilon}$ is:

$$\frac{p(\mathbf{X}|\boldsymbol{\lambda},\sigma^2)p(\mathbf{c}|\mathbf{g},\boldsymbol{\mu},\boldsymbol{\tau})p(K,\mathbf{s}|\phi)p(\mathbf{g}|K,\boldsymbol{\pi})p(\mathbf{c})}{\int_{\mathbf{c}} p(\mathbf{X}|\boldsymbol{\lambda},\sigma^2)p(\mathbf{c}|\mathbf{g},\boldsymbol{\mu},\boldsymbol{\tau})p(K,\mathbf{s}|\phi)p(\mathbf{g}|K,\boldsymbol{\pi})p(\mathbf{c})d\mathbf{c}} = \frac{p(\mathbf{X}|\boldsymbol{\lambda},\sigma^2)p(\mathbf{c}|\mathbf{g},\boldsymbol{\mu},\boldsymbol{\tau})p(\mathbf{c})}{\int_{\mathbf{c}} p(\mathbf{X}|\boldsymbol{\lambda},\sigma^2)p(\mathbf{c}|\mathbf{g},\boldsymbol{\mu},\boldsymbol{\tau})p(\mathbf{c})d\mathbf{c}}$$

Using Eq (5) and Eq (7) and $\boldsymbol{\epsilon}\sim\mathcal{N}(0,\sigma^2)$, the above equation becomes

$$\frac{\prod_{t=1}^{T}p(x_t|\lambda_t,\sigma^2)\times\prod_{k=1}^{K}\mathcal{N}(c_k|\mu_{g_k},\tau_{g_k}^2)}{\int_c(\prod_{t=1}^{T}p(x_t|\lambda_t,\sigma^2)\times\prod_{k=1}^{K}\mathcal{N}(c_k|\mu_{g_k},\tau_{g_k}^2))dc}.$$

The c_k's and the corresponding $\epsilon_{s_k},\cdots,\epsilon_{d_k}$ can be updated one segment at a time with the conditional distribution given by:

$$\frac{\prod_{t=s_k}^{d_k}p(x_t|\lambda_t,\sigma^2)\times\mathcal{N}(c_k|\mu_{g_k},\tau_{g_k}^2)}{\int_c(\prod_{t=s_k}^{d_k}p(x_t|\lambda_t,\sigma^2)\times\mathcal{N}(c_k|\mu_{g_k},\tau_{g_k}^2))dc}.$$

Conditional Posterior Distribution of $\boldsymbol{\psi}$ and $\boldsymbol{\epsilon}$

The conditional posterior distribution of $\boldsymbol{\psi}$ and $\boldsymbol{\epsilon}$ is:

$$\frac{p(\mathbf{X}|\boldsymbol{\lambda},\sigma^2)p(\mathbf{c}|\mathbf{g},\boldsymbol{\mu},\boldsymbol{\tau})p(K,\mathbf{s}|\phi)p(\mathbf{g}|K,\boldsymbol{\pi})p(\boldsymbol{\psi})}{\int_{\boldsymbol{\psi}} p(\mathbf{X}|\boldsymbol{\lambda},\sigma^2)p(\mathbf{c}|\mathbf{g},\boldsymbol{\mu},\boldsymbol{\tau})p(K,\mathbf{s}|\phi)p(\mathbf{g}|K,\boldsymbol{\pi})p(\boldsymbol{\psi})d\boldsymbol{\psi}} = \frac{p(\mathbf{X}|\boldsymbol{\lambda},\sigma^2)p(\boldsymbol{\psi})}{\int_{\boldsymbol{\psi}} p(\mathbf{X}|\boldsymbol{\lambda},\sigma^2)p(\boldsymbol{\psi})d\boldsymbol{\psi}}$$

Using Eq (5) and $\boldsymbol{\epsilon} \sim \mathcal{N}(0,\sigma^2)$, the conditional posterior distribution of $\boldsymbol{\psi}$ and $\boldsymbol{\epsilon}$ given by:

$$\frac{\prod_{t=1}^{T} p(x_t|\lambda_t,\sigma^2) \times p(\psi)}{\int_{\psi}(\prod_{t=1}^{T} p(x_t|\lambda_t,\sigma^2) \times p(\psi))d\psi}.$$

Conditional Posterior Distribution of $\boldsymbol{\theta}$ and $\boldsymbol{\epsilon}$

The conditional posterior distribution of $\boldsymbol{\theta}$ and $\boldsymbol{\epsilon}$ is:

$$\frac{p(\mathbf{X}|\boldsymbol{\lambda},\sigma^2)p(\mathbf{c}|\mathbf{g},\boldsymbol{\mu},\boldsymbol{\tau})p(K,\mathbf{s}|\phi)p(\mathbf{g}|K,\boldsymbol{\pi})p(\boldsymbol{\theta})}{\int_{\boldsymbol{\theta}} p(\mathbf{X}|\boldsymbol{\lambda},\sigma^2)p(\mathbf{c}|\mathbf{g},\boldsymbol{\mu},\boldsymbol{\tau})p(K,\mathbf{s}|\phi)p(\mathbf{g}|K,\boldsymbol{\pi})p(\boldsymbol{\theta})d\boldsymbol{\theta}} = \frac{p(\mathbf{X}|\boldsymbol{\lambda},\sigma^2)p(\boldsymbol{\theta})}{\int_{\boldsymbol{\theta}} p(\mathbf{X}|\boldsymbol{\lambda},\sigma^2)p(\boldsymbol{\theta})d\boldsymbol{\theta}}$$

Using Eq (5) and $\boldsymbol{\epsilon} \sim \mathcal{N}(0,\sigma^2)$, the conditional posterior distribution of $\boldsymbol{\theta}$ and $\boldsymbol{\epsilon}$ given by:

$$\frac{\prod_{t=1}^{T} p(x_t|\lambda_t,\sigma^2) \times p(\theta)}{\int_{\theta}(\prod_{t=1}^{T} p(x_t|\lambda_t,\sigma^2) \times p(\theta))d\theta}.$$
