# Supplementary material for: Bayesian change-point modeling with segmented ARMA model
Source: PLoS One. 2018 Dec 31;13(12):e0208927. doi: 10.1371/journal.pone.0208927 (PMC6312324; doi:10.1371/journal.pone.0208927)
Supplement: S2 Appendix — (PDF) [file pone.0208927.s002.pdf]

Bayesian change-point modeling with segmented ARMA model

Farhana Sadia[1], Sarah Boyd[2], Jonathan M. Keith[1*]

1 School of Mathematical Sciences, Monash University, Clayton, VIC 3800, Australia

2 Faculty of Information Technology, Monash University, Clayton, VIC 3800, Australia

* jonathan.keith@monash.edu

Supporting Information

S2 Appendix. Generalized Gibbs Sampling

This section summarizes the method of the MCMC sampler of Keith *et al.* [1]. In this part, we want to sample from a distribution f over a space $\mathcal{X}$, called the target space.

We define a set $\mathcal{I}$, which will be referred to, in the following, as the index set. We also define $\mathcal{U} \subset \mathcal{I} \times \mathcal{X}$, so that projection of $\mathcal{U}$ onto $\mathcal{X}$ and $\mathcal{I}$ are surjective.

For each $x \in \mathcal{X}$, let $\mathcal{Q}(x)$ be the set $\{(k,z) \in \mathcal{U} : z = x\}$. $\mathcal{Q}(x)$ is a catalogue of the types of transitions available from x.

For every $x \in \mathcal{X}$, we define a transition matrix Q_x on $\mathcal{Q}(x)$. We denote by q_x the density of the distribution which is stationary with respect to Q_x. We denote Q the global transition matrix on $\mathcal{U}$:

$$Q((i,x),(j,y)) = \begin{cases} Q_x((i,x),(j,y)) & \text{for}(j,y) \in \mathcal{Q}(x) \\ 0 & \text{otherwise} \end{cases}$$

For each $(i,x) \in \mathcal{U}$, let $\mathcal{R}(i,x)$ be the set of possible transitions. These sets are required to be a partition of $\mathcal{U}$:

$$(j,y) \in \mathcal{R}(i,x) \Leftrightarrow (i,x) \in \mathcal{R}(j,y)$$

$$\left.\begin{array}{r}(j,y)\in\mathcal{R}(i,x)\\(k,z)\in\mathcal{R}(j,y)\end{array}\right\}\Rightarrow(k,z)\in\mathcal{R}(i,x)$$

We also have $(i,x)\in\mathcal{R}(i,x)$. On $\mathcal{R}(i,x)$, we define a transition matrix $R(i,x)$ as follows :

$$R_{(i,x)}((i,x),(j,y))=\frac{f(y)q_y(j,y)}{\sum_{(k,z)\in\mathcal{R}(i,x)}f(z)q_z(k,z)}$$

We also define a global transition matrix R on $\mathcal{U}$:

$$R((i,x),(j,y))=\begin{cases}R_{(i,x)}((i,x),(j,y)) & \text{for}(j,y)\in\mathcal{R}(i,x)\\ 0 & \text{otherwise}\end{cases}$$

We can generalize this formula by replacing $R((i,x),(j,y))$ with $R((i,x),(j,y))S((i,x),(j,y))$ in the definition of the matrix R, given that $S((i,x),(j,y))=S((j,y),(i,x))$ and adjusting S so that for all $(i,x),\sum_{(j,y)}R((i,x),(j,y))S((i,x),(j,y))=1$. This can be useful to divide the $\mathcal{R}(i,x)$ set into two (or more) subsets.

We consider a Markov chain $\{U_1,U_2,\cdots\}$ on $\mathcal{U}$ with a transition matrix $P=QR$.

$$P((i,x),(j,y))=\sum_{(k,z)\in\mathcal{Q}(x)\cap\mathcal{R}(j,y))}Q((i,x),(k,z))R((k,z),(j,y))$$

Let μ be the distribution defined by $\mu(i,x)=f(x)q_x(i,x)$.

We have

$$\mu(i,x)R((i,x),(j,y))=\begin{cases}\frac{f(x)q_x(i,x)f(y)q_y(j,y)}{\sum_{(k,z)\in\mathcal{R}(i,x)}f(z)q_z(k,z)} & \text{if}(j,y)\in\mathcal{R}(i,x)\\ 0 & \text{otherwise}\end{cases}$$

$$\mu(j,y)R((j,y),(i,x))=\begin{cases}\frac{f(x)q_x(i,x)f(y)q_y(j,y)}{\sum_{(k,z)\in\mathcal{R}(j,y)}f(z)q_z(k,z)} & \text{if}(i,x)\in\mathcal{R}(i,x)\\ 0 & \text{otherwise}\end{cases}$$

We know that $(j,y)\in\mathcal{R}(i,x),(k,z)\in\mathcal{R}(j,y)\Rightarrow(k,z)\in\mathcal{R}(i,x)$, so

$$\mu(i,x)R((i,x),(j,y))=\mu(j,y)R((j,y),(i,x))$$

(note that if $S((i,x),(j,y)) = S((j,y),(i,x))$ and

$$\sum_{(i,x)\in\mathcal{U}} \mu(i,x)Q((i,x),(j,y)) = \sum_{(i,y)\in\mathcal{Q}(y)} f(y)q_y(i)Q((i,y),(j,y))$$
$$= f(y)q_y(j) = \mu(j,y)$$

So, μ is stationary with respect to Q and to R and with respect to P. If P is irreducible and aperiodic, μ is the limiting distribution of the process P. So, we have the generalized Gibbs sampler :

1. Q-step : Given $U_n = (i,x)$, generate $V \in \mathcal{Q}(x)$ by drawing from the distribution with density $Q((i,x),.)$.

2. R-step : Given $V = (j,y)$, generate $W \in \mathcal{R}(j,y)$ by drawing from the distribution with density $R((j,y),.)$.

3. Let $U_{n+1} = W$.

Now we redefine R for the generalisation of the GGS sampler by including Metropolis' sampler, Hastings' generalisations and the reversible jump sampler, that is,

$$R((i,x),(j,y)) = \begin{cases} \frac{s((i,x),(j,y))f(y)q_y(j)}{\sum_{(k,z)\in\mathcal{R}(i,x))} f(z)q_z(k)} & \text{if } (j,y) \in R((i,x))\backslash\{(i,x)\} \\ 1 - \sum_{(k,z)\in\mathcal{R}(i,x))\backslash\{(i,x)\}} R((i,x),(k,z)) & \text{if } (j,y) = (i,x) \\ 0 & \text{otherwise} \end{cases}$$

Here s is a non-negative and symmetric function such that,

$$\sum_{w\in\mathcal{R}(i,x)\backslash\{(i,x)\}} R((i,x),(k,z)) \le 1$$

In case of involving non-denumerable target space, a measure ζ exits on the set $\mathcal{R} = \{\mathcal{R}(i,x) : (i,x) \in \mathcal{U}\}$ and measures η_r on r for each $r \in \mathcal{R}$ such that

$$\xi(A) = \int_{\mathcal{R}} \eta_r(A \cap r)d\zeta(r)$$

where, ξ is a reference measure on $\mathcal{U}$. Now, we can define $R_{(i,x)}((i,x),.)$ to be the density with respect to η_r on $r = \mathcal{R}(i,x)$ given by

$$R_{(i,x)}((i,x),(j,y)) = \frac{f(y)q_y(j,y)}{\int_r f(z)q_z(k,z)d\eta_r(k,z)}$$

We can also define $R_{(i,x)}((i,x),.)$ to be the density with respect to η_r on $r\backslash\{(i,x)\}$ and assign probability mass to (i,x) given by

$$\begin{aligned} R_{(i,x)}((i,x),(j,y)) &= \frac{s((i,x),(j,y))f(y)q_y(j,y)}{\int_r f(z)q_z(k,z)d\eta_r(k,z)} \\ &= 1 - \frac{\int_{r\backslash(i,x)} s((i,x),(j,y))f(y)q_y(j,y)d\eta_r(j,y)}{\int_r f(z)q_z(k,z)d\eta_r(k,z)} \end{aligned}$$

The function s must satisfy $\frac{\int_{r\backslash(i,x)} s((i,x),(j,y))f(y)q_y(j,y)d\eta_r(j,y)}{\int_r f(z)q_z(k,z)d\eta_r(k,z)} \leq 1$.

In case of reversible jump MCMC sampler instance of the GGS, we define a transition density function $Q_x((m_0,y_0,x_0),(m,y,x)) = q_x(m,y,x)$ in Q-step, where $q_x(m,y,x) = \sigma_x(m)A_m(x,y)$. Here, m is a move-type; $m \in \mathcal{M}$ ($\mathcal{M}$ is the countable set of move-types), $\sigma_x(m)$ is the probability of selecting move-type m at proposed new element x and $A_m(x,y)$ is the density for move-type m at x.

In R-step, the density of μ is $\sigma_x(m)f_m(x,y)$ at (m,y,x) with respect to ξ and we define a partition of $\mathcal{U}$ which consists of $\mathcal{R}(m,y,x) = \{(m,y,x),(m,x,y)\}$ for all $(m,y,x) \in \mathcal{U}$. Here,

$$s((m,y,x),(m,x,y)) = \min\left(1 + \frac{\sigma_m(x)f_m(x,y)}{\sigma_m(y)f_m(y,x)}, 1 + \frac{\sigma_m(y)f_m(y,x)}{\sigma_m(x)f_m(x,y)}\right)$$

This s matrix gives the transition matrix

$$R_{(i,x)}((i,x),(j,y)) = \begin{cases} \alpha_m(x,y) & \text{if } (j,y) = (i,x) \equiv (m,y,x) \\ 1 - \alpha_m(x,y) & \text{if } (j,y) = (m,y,x) \end{cases}$$

where,

$$\alpha_m(x,y) = \min\left(1, \frac{\sigma_m(y)f_m(y,x)}{\sigma_m(x)f_m(x,y)}\right).$$

Here, new element(m,y,x) is selected with the transition matrix R which is equivalent to accepting the proposed y with probability $\alpha_m(x,y)$.

References

1. Keith JM, Kroese DP, Bryant D. A generalized Markov sampler. Methodology and Computing in Applied Probability. 2004;6(1):29–53.
