# Supplementary material for: Bayesian change-point modeling with segmented ARMA model
Source: PLoS One. 2018 Dec 31;13(12):e0208927. doi: 10.1371/journal.pone.0208927 (PMC6312324; doi:10.1371/journal.pone.0208927)
Supplement: S3 Appendix — (PDF) [file pone.0208927.s003.pdf]

Bayesian change-point modeling with segmented ARMA model

Farhana Sadia[1], Sarah Boyd[2], Jonathan M. Keith[1*]

1 School of Mathematical Sciences, Monash University, Clayton, VIC 3800, Australia

2 Faculty of Information Technology, Monash University, Clayton, VIC 3800, Australia

* jonathan.keith@monash.edu

S3 Appendix. Supplementary Material A

To test our method, we generated two simulated data sets. In both data sets, we generated 20 time series of 100 observations, each with a different segment mean, from the autoregressive moving average (ARMA (1,1)) model with parameter values $\psi = 0.22$ and $\theta = 0 : 60$. The two data sets differed in the value of the parameter σ^2 used to generate the data: in the first we used $\sigma^2 = 0.96$ and in the second we used $\sigma^2 = 0.5$. We applied our methods for the segmented ARMA model and segmented AR model to these data and compared the location of change points and number of change points found by these two models.

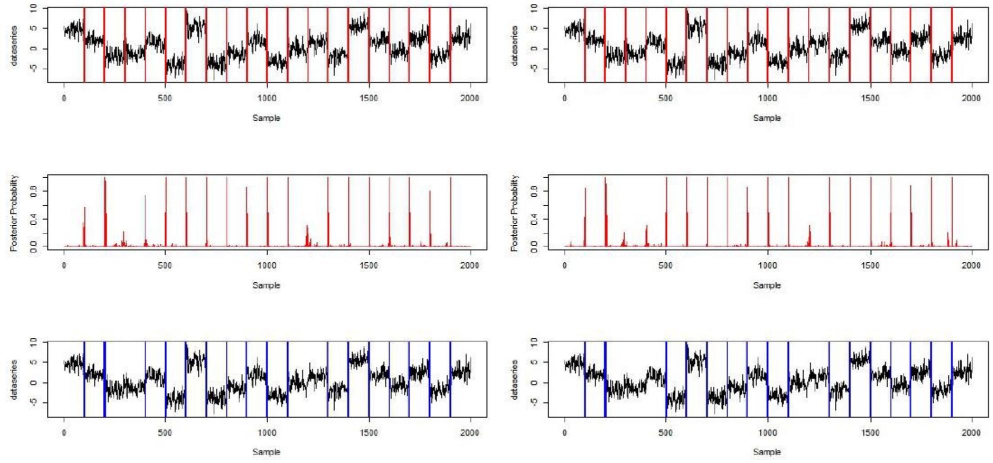

(a) Segmented ARMA with $\sigma^2 = 0.96$ (b) Segmented AR with $\sigma^2 = 0.96$

S3 Fig 1. Segmented ARMA model and segmented AR model with $\sigma^2 = 0.96$

The top panels of S3 Fig 1 present the simulated signal for the first data set ($\sigma^2 = 0.96$) with the true change-points marked as red vertical lines. The middle plots show the posterior probabilities of occurrence of change-point locations and the bottom plots show the estimated change-point locations using a threshold (0.5) in the posterior probabilities of occurrence of change-points. If we compare the above plots, it is clear that the segmented ARMA and segmented AR models find almost the same number of change points and locations. But in some locations, the segmented ARMA model gives higher posterior probability than the segmented AR model. When we apply a threshold (the posterior probability of occurrence of change-points is greater than 0.5), the segmented ARMA model identifies 17 change-points out of 19 true change-points whereas the segmented AR model identifies 16 change-points.

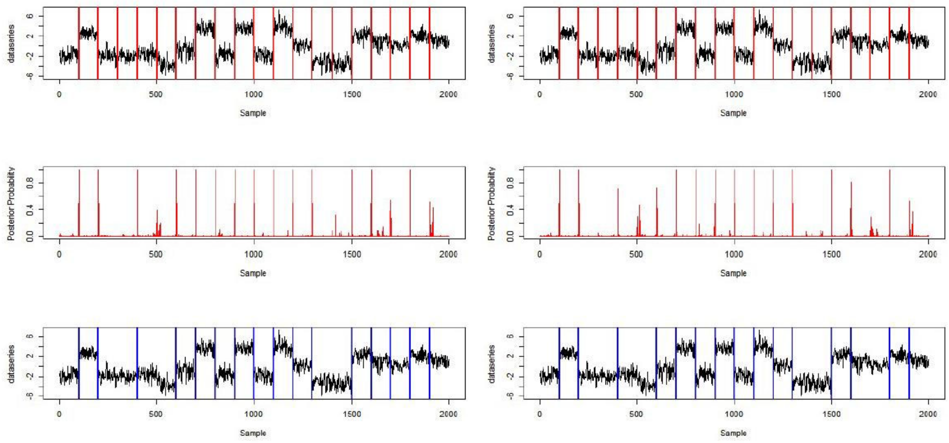

(a) Segmented ARMA with $\sigma^2 = 0.5$ (b) Segmented AR with $\sigma^2 = 0.5$

S3 Fig 2. Segmented ARMA model and segmented AR model with $\sigma^2 = 0.5$

Results for the second simulated data set ($\sigma^2 = 0.5$) are shown in S3 Fig 2. These plots also demonstrate that the ARMA and AR models find the same number of change-points and the same locations, but in some locations the posterior probability of occurrence of change-points is lower using the segmented AR model than the segmented ARMA model. The bottom panel of the segmented AR plot indicates 15 change-points were identified out of 19 but the segmented ARMA model identifies 16 change-points. These results suggest that the segmented ARMA model identifies significant change-points with higher probability of occurrence than the AR model, when the data are generated using an ARMA model, regardless of the value of σ^2.
