# Supplementary material for: Bayesian change-point modeling with segmented ARMA model
Source: PLoS One. 2018 Dec 31;13(12):e0208927. doi: 10.1371/journal.pone.0208927 (PMC6312324; doi:10.1371/journal.pone.0208927)
Supplement: S4 Appendix — (PDF) [file pone.0208927.s004.pdf]

Bayesian change-point modeling with segmented ARMA model

Farhana Sadia[1], Sarah Boyd[2], Jonathan M. Keith[1*]

1 School of Mathematical Sciences, Monash University, Clayton, VIC 3800, Australia

2 Faculty of Information Technology, Monash University, Clayton, VIC 3800, Australia

* jonathan.keith@monash.edu

Supporting Information

S4 Appendix. Supplementary Material B

Our computational algorithm is not sensitive to the choice of prior for ϕ. The posterior distribution of ϕ is a beta distribution that is scarcely changed by the choice of prior, as the following graphs indicate. The top panel in S4 Fig 1 below shows five low information prior distributions for ϕ, with different means and variances. S4 Fig 2 show the posterior distributions obtained using each of the five priors. Despite the large discrepancies in the prior information, the posterior distributions are indistinguishable.

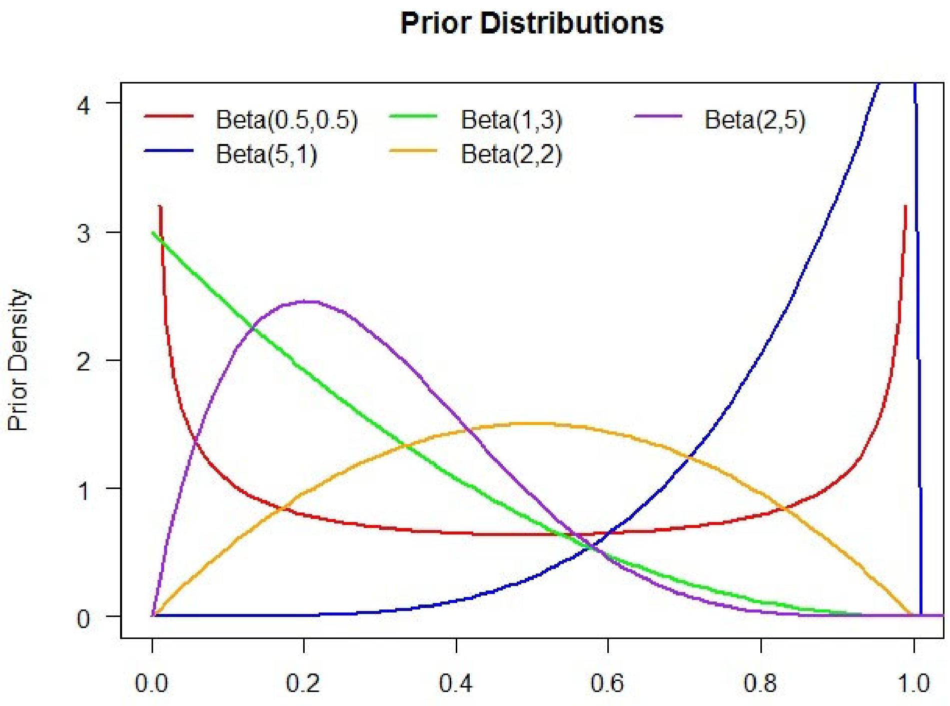

S4 Fig 1. Different prior distributions for ϕ

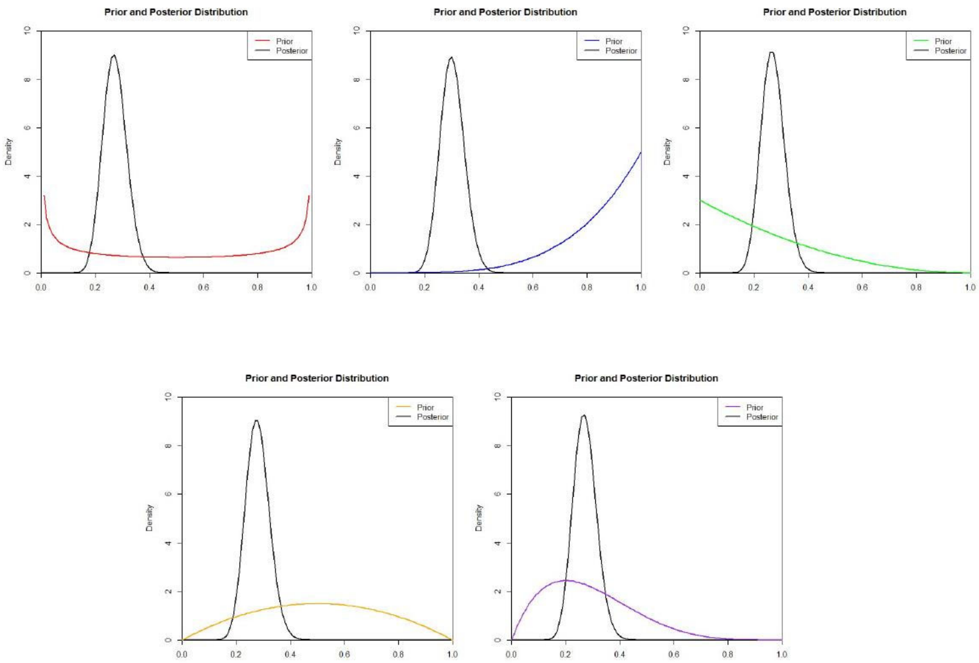

S4 Fig 2. Posterior distributions for different prior distributions
